# Microglia Sirt6 modulates the transcriptional activity of NRF2 to ameliorate high-fat diet-induced obesity

**DOI:** 10.1186/s10020-023-00676-9

**Published:** 2023-08-15

**Authors:** Xiaoxia Xiao, Huiling Hu, Yadi Zhong, Yingjian Chen, Kaijia Tang, Zhisen Pan, Jiawen Huang, Xiaoying Yang, Qi Wang, Yong Gao

**Affiliations:** 1 Science and Technology Innovation Center, Guangzhou University of Chinese Medicine, Guangzhou, 510006 China; 2 Department of Clinical Laboratory, Sun Yat-Sen Memorial Hospital, Sun Yat-Sen University, Guangzhou, 510289 China; 3 Guangdong Provincial Key Laboratory of Malignant Tumor Epigenetics and Gene Regulation, Sun Yat-Sen Memorial Hospital, Sun Yat-Sen University, Guangzhou, 510289 China; 4 First Affiliated Hospital, Guangzhou University of Chinese Medicine, Guangzhou, 510006 China; 5 Jiangsu Key Laboratory of Immunity and Metabolism, Department of Pathogen Biology and Immunology, Xuzhou Medical University, Xuzhou, 221004 Jiangsu China

**Keywords:** Sirt6, Microglial activation, Hypothalamic inflammation, NRF2, Obesity

## Abstract

**Background:**

Microglia play a pivotal role in neuroinflammation, while obesity triggers hypothalamic microglia activation and inflammation. Sirt6 is an important regulator of energy metabolism in many peripheral tissues and hypothalamic anorexic neurons. However, the exact mechanism for microglia Sirt6 in controlling high-fat diet-induced obesity remain unknown.

**Methods:**

Microglia Sirt6 expression levels under various nutritional conditions were measured in the hypothalamus of mice. Also, microglia Sirt6-deficient mice were provided various diets to monitor metabolic changes and hypothalamic inflammatory response. Besides, RNA-seq and Co-IP of microglia with Sirt6 alterations were conducted to further investigate the detailed mechanism by which Sirt6 modulated microglia activity.

**Results:**

We found that Sirt6 was downregulated in hypothalamic microglia in mice given a high-fat diet (HFD). Additionally, knockout of microglia Sirt6 exacerbated high-fat diet-induced hypothalamic microglial activation and inflammation. As a result, mice were more prone to obesity, exhibiting a decrease in energy expenditure, impaired glucose tolerance, insulin and leptin resistance, and increased food intake. In vitro, Sirt6 overexpression in BV2 cells displayed protective effects against oleic acid and palmitic acid treatment-derived inflammatory response. Mechanically, Sirt6 deacetylated and stabilised NRF2 to increase the expression of anti-oxidative genes and defend against reactive oxygen species overload. Pharmacological inhibition of NRF2 eliminated the beneficial modulating effects of Sirt6 on microglial activity.

**Conclusion:**

Collectively, our results revealed that microglial Sirt6 was a primary contributor of microglial activation in the central regulation of obesity. Thus, microglial Sirt6 may be an important therapeutic target for obesity.

**Supplementary Information:**

The online version contains supplementary material available at 10.1186/s10020-023-00676-9.

## Introduction

Achieving an energy balance in the body involves numerous biochemical reactions and hormonal signalling pathways. However, a persistent positive energy balance leads to common metabolic-related diseases such as obesity (Nadal et al. 2017). The hypothalamus is involved in the regulation of energy metabolism. Additionally, specific areas of the hypothalamus, such as the paraventricular nucleus (PVN), perifornical area (PFA), lateral hypothalamic area (LHA), and arcuate nucleus (ARC), synergistically regulate energy metabolism by integrating neural, nutritional, and hormonal signals (Schwartz et al. 2000). Microglia are the immune-competent cells of the brain and their activation may result in abnormal neurons in the hypothalamus, causing glucose and lipid metabolism disorders in the body. The mechanism of this process involves activated hypothalamic microglia producing a variety of pro-inflammatory cytokines that influence metabolism by inducing changes in neuronal activity (Reis et al. 2015; Valdearcos et al. 2014). HFD exposure triggers microglial activation and hypothalamic inflammation, which is manifested by altered microglial morphology (transformed to an M1-like pro-inflammatory phenotype), activation of inflammatory signalling, and increased expression of inflammatory cytokines (Mendes et al. 2018; André et al. 2017). Furthermore, hypothalamic inflammation occurs before significant weight gain (Thaler et al. 2012), suggesting that it may be an upstream event in diet-induced obesity (DIO). Drug depletion of microglia limits diet-induced weight gain. Conversely, specific activation of microglia induces food intake and subsequent weight gain in mice (Valdearcos et al. 2017). These findings support the potential of microglia as central energy regulators. However, the extent to which inflammatory activation of microglia affects metabolism is uncertain, and further investigation into the role of microglia in coordinating metabolism is necessary.

Sirtuin 6 (Sirt6) is a member of the Sirtuin family of NAD+-dependent enzymes, with three forms of enzymatic activity: deacetylase, adenosine diphosphotransferase, and defat acylase. As a chromatin-associated nuclear protein, Sirt6 is highly expressed in the central nervous system (CNS) and it reduces inflammatory damage caused by microglial activation. For instance, endothelial Sirt6 plays a protective role in cerebral ischemia/reperfusion injury (Liberale et al. 2020). Additionally, the pharmacological activation of Sirt6 ameliorates neuroinflammation and attenuates brain injury in mice with ischemic stroke (He et al. 2021). As an NAD+-dependent enzyme, one primary function of Sirt6 is to regulate energy metabolism in the body. Initial studies found that Sirt6-deficient mice exhibited severe metabolic defects (Mostoslavsky et al. 2006). Subsequent studies established that Sirt6 transgenic mice were resistant to high-fat diet-induced obesity, manifested by less fat accumulation (Kanfi et al. 2010). Similarly, adipose-specific Sirt6 knockout mice and myeloid-specific Sirt6 knockout mice are more susceptible to DIO (Yao et al. 2017; Lee et al. 2017). Recent studies have shown that Sirt6 may regulate metabolism in relation to its anti-inflammatory effect. Fat-specific Sirt6 deficiency promotes adipose tissue inflammatory response, which contributes to insulin resistance (Kuang et al. 2017). Also, myeloid-specific Sirt6 deletion promotes M1-type pro-inflammatory macrophage expression in mice. As a result, mice exhibit increased hepatic tissue inflammation and aggravated hepatic steatosis (Lee et al. 2017). Currently, the role of Sirt6 in diet-induced obesity-related hypothalamic inflammation has not been reported. The mechanism by which microglia Sirt6 coordinates DIO also remains to be explored.

Transcription factor NRF2 (Nuclear Factor Erythroid 2-related Factor 2) is one of the main regulators of the antioxidant defence system in the body. Normally, NRF2 associates with Kelch-like ECH-associated Protein 1 (Keap1) in the cytoplasm and is degraded by the ubiquitinase Cullin3. During oxidative stress, NRF2 dissociates from the complex and translocates to the nucleus, where it binds to DNA at the antioxidant response elements (ARE) position, thereby promoting the expression of downstream antioxidant enzymes (Silva-Islas and Maldonado 2018). Activation of NRF2 is associated with the amelioration of oxidative stress and reduction of neuroinflammatory responses. In neurodegenerative diseases associated with oxidative homeostasis, such as Parkinson’s disease, promoting the NRF2 antioxidant pathway improves mitochondrial and neuronal function and protects the dopaminergic neurons of mice from oxidative damage (Martín-Montañez et al. 2021). Besides, NRF2 deficiency exacerbates gliosis and neuroinflammatory responses in mouse models with combined tauopathy and amyloidopathy (Rojo et al. 2018). Additionally, enhancing the p21-NRF2 axis of microglia ameliorates neurodegeneration by suppressing neuroinflammation (Nakano-Kobayashi et al. 2020). The potential of NRF2 in metabolic regulation has gradually emerged in recent years. NRF2 has been reported to prevent oxidative stress and restore insulin secretion in mouse pancreatic β-cells in the context of reactive species damage (Yagishita et al. 2014). NRF2 also improves insulin and leptin resistance in mice by reducing oxidative damage in the mouse hypothalamus (Yagishita et al. 2017). These findings highlight the role of NRF2 in the CNS and metabolic-related diseases. However, the exact role of NRF2 and its upstream and downstream effectors in metabolic regulation still requires further investigation.

The results from our study reveal that Sirt6 is involved in the regulation of M1/M2 phenotypic transition in microglia, and further confirm that hypothalamic microglia Sirt6 plays a critical role in long-term high-fat diet-induced obesity. Furthermore, we found that Sirt6 maintains its stability by deacetylating NRF2, thereby achieving the purpose of anti-inflammation and anti-oxidation and ultimately regulating the damage caused by a HFD.

## Materials and methods

### Animal and experimental designs

Male C57BL/6J mice (6–8 weeks old) were provided by the Animal Experiment Centre of Guangzhou University of Chinese Medicine and Cx3cr1-Cre mice were purchased from Cyagen. Additionally, SIRT6^flox/flox^ mice (hereafter referred to as SIRT6^fl/fl^) that were originally purchased from the Jackson Laboratory (Bar Harbor, ME, USA) were donated by the West China Hospital of Sichuan University (Zhang et al. 2021). Microglia Sirt6 conditional knockout mice (hereafter referred to as SIRT6^Mic−/−^) were produced by mating SIRT6^fl/fl^ mice with Cx3cr1-Cre mice. All animals were housed under standard laboratory conditions with a 12-h light/dark cycle. Mice were allowed access to a standard chow diet (SD) or HFD (research diet, D12492, USA) for 12 weeks. Besides, all animals had free access to food and water throughout the experiment unless otherwise stated. All animal handling complied with the guidelines of the Animal Ethics Committee of Guangzhou University of Chinese Medicine, and the experimental protocols were approved.

At the end of the study, the mice were anesthetized by Pentobarbital sodium salt (57-33-0, sigma). Next, the mice were euthanized by cardiac perfusion or cervical dislocation. Part of the tissue was fixed in 4% paraformaldehyde solution and part was kept in a freezer at -80 ℃. The collected blood was centrifuged to obtain serum, which was also stored at -80 ℃. The number of animals used is detailed in the figure legends.

### Haematoxylin and eosin (HE) staining

Liver and adipose tissue samples were fixed with 4% paraformaldehyde, dehydrated using different concentration gradients of ethanol, embedded in paraffin, and cut into 4 μm thick sections. Sections were deparaffinised, stained with haematoxylin and eosin (HE), dehydrated, and mounted with neutral gum. The staining of the tissue sections was imaged under a Nikon microscope (Eclipse, 80i).

### Oil red O staining

Liver tissue was fixed with 4% paraformaldehyde, dehydrated with 30% sucrose solution, embedded in OCT gel, and cut into 7 μm thick sections using a Leica CM1950 frozen microtome. Sections were stained with oil red, differentiated with 60% isopropanol, further stained with haematoxylin, and then finally mounted. Images were collected using a Nikon microscope.

### Fat assay of mice

Mice after 12 weeks of the HFD were subjuected to micro-CT scannning. Subsequently, the brown fat, subcutaneous fat, and epididymal fat of the mice were analyzed and reconstructed using the software.

### Serum biochemical test

Serum insulin was measured using a mouse insulin (INS) ELISA kit (RX202485M, Ruixin Biotech) and calculated according to the directions. Serum norepinephrine was determined with a mouse norepinephrine ELISA Kit and calculated according to the manufacturer’s instructions.

### Glucose tolerance test (GTT), insulin tolerance test (ITT), and pyruvate tolerance test (PTT)

Following our previously reported methods (Sun et al. 2021), mice were subjected to GTT, ITT and PTT tests after 12 weeks of the HFD. Briefly, for GTT, after fasting for 16 h, mice were injected intraperitoneally with glucose (1.5 g/kg) and the tail vein blood glucose was measured at six time points (0, 15, 30, 60, 90, 120 min) with a blood glucose meter. As for ITT, mice were injected intraperitoneally with human insulin (0.75 U/kg) after fasting for 4 h, and the tail vein blood glucose was measured at the same time points as for GTT. Regarding PTT, mice were injected intraperitoneally with sodium pyruvate (1 g/kg) after fasting for 16 h, and the tail vein blood glucose was measured at the same time points as the other two tests.

### Leptin function and sensitivity studies

Murine Leptin (AF-450-31-1MG, PeproTech) was intraperitoneally injected into SIRT6^fl/fl^ and SIRT6^Mic−/−^ mice. Next, the food intake and body weight of the mice were measured. Additionally, SIRT6^fl/fl^ and SIRT6^Mic−/−^ mice were perfused 45 min after leptin injection and the expression of P-STAT3 (Ser727) in mouse brain tissue was detected through immunofluorescence.

### Immunohistochemistry

Brown adipose tissue sections were obtained in the same manner as for HE staining, as described above. The sections were deparaffinised and rehydrated with xylene and different concentrations of ethanol. Next, 3% hydrogen peroxide was used to quench endogenous peroxidase and antigen retrieval was performed in citrate buffer. After blocking with 10% goat serum, the primary antibody UCP1 (P25874, CUSABIO) was added, the mixture was kept overnight at 4 ℃, and then it was incubated with HRP-conjugated secondary antibody for 30 min at room temperature. After DAB working solution staining and haematoxylin counterstaining, the samples were further dehydrated with increasing concentrations of ethanol and finally mounted. Image information was collected under the microscope.

### Cold exposure test

Following our previous method (Tang et al. 2020), mice after 12 weeks of the HFD were transferred from room temperature to 4 ℃ refrigerator (BIOBASE, BYC-1000) and their body temperatures were measured at 0 h, 2 h, 4 h, and 6 h.

### Metabolic parameter measurements

Mice after 12 weeks of the HFD were acclimated for 48 h in advance and energy expenditure was measured using a comprehensive laboratory animal monitoring system (CLAMS). During the experiment, the surrounding environment was kept quiet and the mice were provided sufficient water and food.

### Isolation and culture of mouse primary microglia

Mice after 24 weeks of the HFD were used to isolate primary microglia. The mice were sacrificed by cervical dislocation, and their brain tissue was isolated and placed in PBS solution. The tissue masses were adopted for preparing cell suspension through mechanical grinding of Dounce homogenizer, and then the cell suspension was filtrated by a 70 μm (200 mesh) cell strainer for debris removal. After 7 min of 300 g centrifugation at a low speed, the supernatant was discarded, and the cells were collected. Percoll (17-0891-09, GE Healthcare) was mixed in 10×PBS to make solutions at three different concentrations, i.e., 30%, 37% and 70%. The collected cells were transferred to 37% Percoll solution. In a 15 ml centrifuge tube, 4 ml of 70% Percoll solution, 4 ml of 37% Percoll solution containing cells, and 4 ml of 30% Percoll solution were add in sequence, and at last, 2 ml of PBS was added. The mixture underwent 40 min of 300 g centrifugation at 18 ℃. After the centrifugation, the cells were collected from the 37% and 70% gradient interfaces and washed twice with 6 ml PBS. At last, the cells were collected for Quantitative Real-Time PCR.

For the one-day-old neonatal mice, the brain of mice was collected using microsurgical forceps. The tissue was digested with trypsin (C0207, Beyotime), centrifuged, resuspended in complete medium and filtered through a 70 μm cell strainer to obtain mixed glial cells. The cells were cultured in an incubator and the medium was changed every 3 days. After 15 days of culture, the cells appeared stratified. Microglia are attach to the surface of astrocytes. Next, the microglia were shaken down using a shaker, with operating conditions of 37 ℃ and 200 rpm, for 2 h. The collected microglia were resuspended in complete medium to continue culturing in the incubator. At last, the cells were collected for Western blot analysis.

### BV2 cells culture

BV2 cells (Wuhan University Cell Bank, China) were cultured in DMEM medium containing 10% foetal bovine serum at standard cell culture conditions (5% CO2, 95% air). The cells were operated for no more than 10 generations.

### BV2 cells treatment

Oleic acid (OA) may stimulate the formation of lipid droplets through activating FFAR4, a long-chain fatty acid receptor (Rohwedder et al. 2014; Chausse et al. 2019), while palmitic acid (PA) is a major contributor to diet-induced neuroinflammation (Chausse et al. 2019; Valdearcos et al. 2015). Here, we utilized OA&PA combination to treat the cells, thus simulating the effects of a high-fat diet stimulation. In the overexpression experiment, adenoviruses Ad-Sirt6 (Obio Technology, Shanghai, China) and Ad-GFP were used for 12 h infection of BV2 cells, and in the knockdown experiment, adenoviruses Ad-shSirt6 (Obio Technology, Shanghai, China) and Ad-shGFP were adopted for 12 h infection of BV2 cells. Subsequently, BV2 cells were cultured for 24 h in a fresh medium containing OA and PA, and to inhibit NRF2, BV2 cells were treated with ML-385 (846557-71-9, selleck) for 12 h. At last, the cells were collected for further analysis.

### Cytokine measurement

The expression levels of tumour necrosis factor alpha (TNF-α), interleukin 6 (IL-6), and interleukin 1β (IL-1β) in the culture supernatant were measured using the mouse TNF-α ELISA kit (JL10484, Jianglai, Shanghai, China), mouse IL-6 ELISA kit (JL20268, Jianglai, Shanghai, China), and mouse IL-1β ELISA kit (JL18442, Jianglai, Shanghai, China), respectively.

### Co-immunoprecipitation (Co-IP) assay

Adenoviruses Ad-Sirt6 and Ad-GFP were transfected into BV2 cells, then the cells were collected to extract proteins. Experiments were performed using protein A + G agarose (Fast Flow, P2012, Beyotime). Specifically, protein A + G agarose and normal IgG of the same species as the IgG used for immunoprecipitation were added to remove non-specific binding of the protein sample. After the samples were incubated overnight with Sirt6 (67510-1-Ig, Proteintech) or NRF2 (16396-1-AP, Proteintech), protein A + G agarose was added. The mixture was shaken slowly at 4 ℃ for 3 h and then centrifuged and denatured. Subsequent western blot experiments were performed using Sirt6 (13572-1-AP, Proteintech) and NRF2 (ab89443, Abcam) antibodies.

For the acetylation assays, the cells were lysed in lysis buffer containing a protease inhibitor, phosphatase inhibitors, and acetylatase inhibitor. Protein A + G agarose and IgG were added to remove non-specific binding of the protein sample. After the samples were incubated overnight with NRF2 (ab89443, Abcam), protein A + G agarose was added. Next, the mixture was shaken slowly at 4 ℃ for 3 h, then it was centrifuged and denatured. Subsequent western blot experiments were performed using Pan Acetyl-Lysine Rabbit pAb (A2391, ABclonal) to detect the acetylation levels of NRF2.

### Intracellular reactive oxygen species (ROS) assay

After the preparation of the cell samples, a fluorescent probe DHE (38483-26-0, KeyGEN BioTECH) was used for ROS determination. For this process, 10 µM DHE was added to the cells, which were then incubated at 37 ℃ for 20 min in the dark and washed with PBS. Finally, fluorescence images were taken with a LEICA DMi8 fluorescent inverted microscope.

### Immunofluorescence

Brain tissue was fixed with 4% paraformaldehyde for 48 h, dehydrated with 30% sucrose solution, and then embedded in OCT gel. The tissue was cut into 30 μm thick coronal sections with a Leica CM1950 frozen microtome. Sections were then treated with 1% Triton™ X-100 (T8787, Sigma-Aldrich) and 5% BSA (A8020, Solarbio) for 1 h at room temperature and subsequently incubated overnight at 4 ℃ with the following primary antibodies: Anti-Iba1 antibody (ab5076, Abcam), Sirt6 (13572-1-AP, Proteintech), TNF-α (A11534, ABclonal), and P-STAT3 (11,046, SAB). Donkey Anti-Rabbit IgG H&L (Alexa Fluor® 488, ab150073, Abcam) was used for single staining, while Donkey Anti-Rabbit IgG H&L and Donkey Anti-Goat IgG H&L (Alexa Fluor® 594, ab150132, Abcam) were employed for double staining. After the antibody was incubated for 1 h at room temperature, the slides were mounted with DAPI-containing mounting medium (S2110, Solarbio). Finally, fluorescence images were acquired using an SP8 Leica confocal fluorescence microscope (TCS-SP8).

For cellular immunofluorescence, cell samples were fixed with 4% paraformaldehyde for 20 min at room temperature, treated with 0.3% Triton™ X-100 for 15 min, and blocked with 5% BSA for 1 h. The main primary antibodies used were: CD68 (BA3638, Boster), TNF-α (A11534, ABclonal), and NRF2 (16396-1-AP, Proteintech). Besides, Alexa Fluor 594-conjugated AffiniPure Goat Anti-Rabbit IgG (H + L) (AS074, ABclonal) was selected as the secondary antibody. Images were collected using a Nikon fluorescence microscope.

### Quantitative real-time PCR

Total RNA was extracted using MagicZol reagent and reverse transcribed using the 5X All-In-One RT MasterMix kit (G490, abm). Besides, 2X Universal SYBR Green Fast qPCR Mix kit (RK21204, ABclonal) was used for quantitative real-time PCR. Also, β-actin was used as an internal control and 2^ΔΔCt^ was applied to calculate the relative expression of the target gene. The analyzed genes and the primer sequences are listed in Table 1.

**Table 1:** Primer sequences used for the qPCR analysis

Gene	Forward Primer (5ʹ‑3ʹ)	Reverse Primer (5ʹ‑3ʹ)
*β-actin*	GGCTGTATTCCCCTCCATCG	CCAGTTGGTAACAATGCCATGT
*Sirt6*	ATGTCGGTGAATTATGCAGCA	GCTGGAGGACTGCCACATTA
*Ptp1b*	GGAACTGGGCGGCTATTTACC	CAAAAGGGCTGACATCTCGGT
*Lepr*	TGGTCCCAGCAGCTATGGT	ACCCAGAGAAGTTAGCACTGT
*Pi3k*	ACACCACGGTTTGGACTATGG	GGCTACAGTAGTGGGCTTGG
*Jak2*	TTGTGGTATTACGCCTGTGTATC	ATGCCTGGTTGACTCGTCTAT
*Socs3*	ATGGTCACCCACAGCAAGTTT	TCCAGTAGAATCCGCTCTCCT
*Tnf-α*	CCCTCACACTCAGATCATCTTCT	GCTACGACGTGGGCTACAG
*Il-6*	TAGTCCTTCCTACCCCAATTTCC	TTGGTCCTTAGCCACTCCTTC
*Il-1β*	GCAACTGTTCCTGAACTCAACT	ATCTTTTGGGGTCCGTCAACT
*Cd16*	CAGAATGCACACTCTGGAAGC	GGGTCCCTTCGCACATCAG
*Cd86*	TGTTTCCGTGGAGACGCAAG	TTGAGCCTTTGTAAATGGGCA
*Ym1/2*	CAGGGTAATGAGTGGGTTGG	CACGGCACCTCCTAAATTGT

### Western blot analysis

Total protein was extracted from primary microglia. The samples were lysed in RIPA buffer (P0013C, Beyotime) containing protease and phosphatase inhibitors. The BCA protein concentration assay kit (BL521A, Biosharp) was used for quantification. The protein was denatured at 100 ℃, loaded on a 10% SDS-PAGE gel (P0012A, Beyotime), separated by electrophoresis, and transferred to a polyvinylidene fluoride PVDF membrane (IPVH00010, Immobilon) by wet transfer. After blocking with 5% milk for 1 h at room temperature, the Sirt6 (13572-1-AP, Proteintech) and β-actin (AP0060, Bioword) primary antibodies were used. After secondary antibody (ab205718, Abcam) incubation, detection was performed in a Bio-Rad chemiluminescence imager using the NcmECL ultra-sensitive ECL chemiluminescence kit (P10300, NCM Biotech, Suzhou, China). Quantitative analysis was performed using ImageJ software.

### RNA sequencing

BV2 cells were treated with Adenoviruses Ad-Sirt6 or Ad-GFP for 12 h and then stimulated with OA and PA for 24 h. Total RNA was extracted with MagicZol reagent and RNA quality was detected. After the RNA samples were qualified, a mRNA-seq library was constructed. The library was quality-checked using Qubit 3.0, and PE150 sequencing was performed using the Illumina NovaSeq6000 high-throughput sequencing platform. Next, basic data quality control was performed on the data obtained by sequencing. Specifically, the base sequences (reads) generated by the platform were quality filtered to obtain high-quality clean reads. The clean reads were then aligned to the SILVA database using bowtie2 software to remove rRNA, and the remaining reads were used for subsequent analysis. Then, the rRNA-free reads were aligned to the reference genome using Hisat2 software, and the gene expression level and gene structure were analysed. Additionally, heatmaps were generated using the R package hierarchical clustering algorithm.

### Molecular docking

Since both receptor and ligand proteins are rigid, a rigid docking scheme was used to predict the binding mode between the Sirt6 and NRF2 proteins. From the protein database (https://www.rcsb.org/), the crystal structure of human Sirt6 protein/NAD-dependent deacetylase sirtuin-6 (Uniport ID: Q8N6T7) and nuclear factor erythroid 2-related factor 2/NRF2 (Uniport ID: Q14145) were retrieved with accession numbers 3K35 and 3ZGC, respectively. The initial complex structure was prepared by removing hydrogens, extra side chains, and extra ions, and minimization in PYMOL. Subsequently, rigid docking was achieved using the ZDOCK protocol. In Biovia Discovery Studio Server 2019, the modified protein structure-human Sirt6 protein/NAD-dependent deacetylase sirtuin-6 was set as the receptor protein and nuclear factor erythroid 2-related factor 2/NRF2 was the ligand protein. The docking protein structure was performed by Discovery Studio and the activity of the representative protein was predicted according to the docking results of the natural ligand protein (GFPT1, PDB ID: 2V4M).

### Statistical analysis

Data were presented as mean ± SEM. Statistical analysis was performed using GraphPad Prism version 8.4.0 software. Comparisons between two groups were analysed by the student’s t-test, and a one-way or two-way ANOVA was used for comparison among multiple groups, followed by the Tukey post-hoc test. *P* < 0.05 denoted statistical significance.

## Results

### HFD reduces the expression of Sirt6 in mice hypothalamic microglia

**Fig. 1:**
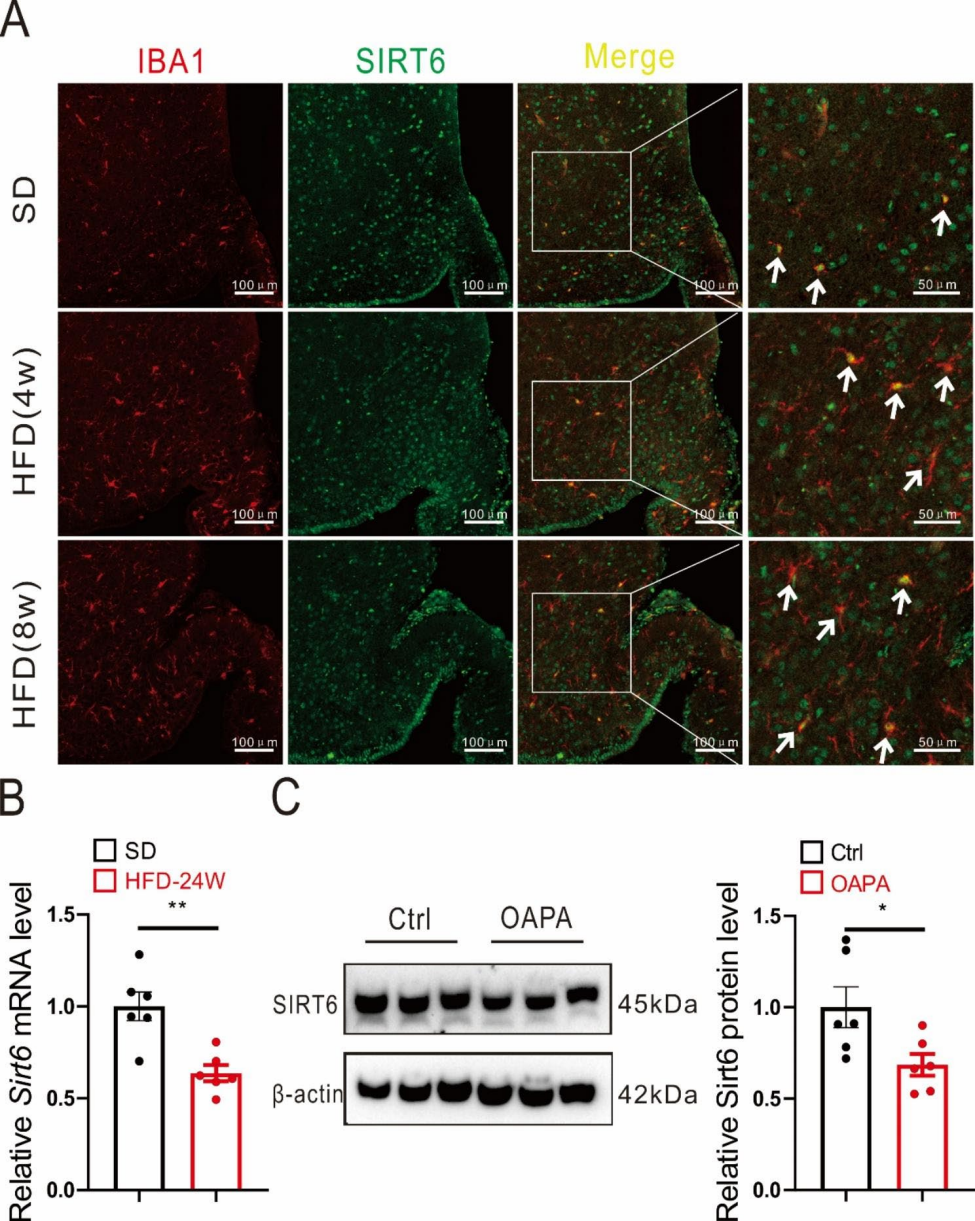
Sirt6 responds to changes in the HFD environment. **A** Confocal images of Iba1 (red) and Sirt6 (green) in the hypothalamus after 4 and 8 weeks of HFD. Arrowheads indicate representative cells showing Ibal-positive cells. Scale bars: 100 μm. **B***Sirt6* mRNA expression level in microglia of male mice on a HFD for 24 weeks. Unpaired t-test. *n* = 6/group. **C** Sirt6 protein expression level in primary microglia of one-day-old neonatal mice after OA&PA treatment for 24 h. Unpaired t-test. *n* = 6/group. Data are presented as mean ± SEM, * *p* < 0.05, ** *p* < 0.01

To assess whether HFD affected the expression of Sirt6 in microglia, confocal experiments were used to observe the hypothalamus of mice exposed to either SD, 4 weeks of HFD, or 8 weeks of HFD. Ibal was selected as a biomarker of microglia. The results showed that HFD triggered hypothalamic microglial activation. Moreover, compared to the SD group, 8 weeks of HFD exposure induced a mild decrease of Sirt6 in the hypothalamic microglia (Fig. 1A). We further measured the mRNA expression level of *Sirt6* in primary microglia of male mice on a HFD for 24 weeks. As expected, mRNA expression level of *Sirt6* were lower in mice on a HFD (Fig. 1B). Similarly, Sirt6 protein expression level was also lower in OA&PA-treated primary microglia of one-day-old neonatal mice (Fig. 1C).

### Sirt6 knockout in microglia exacerbates high-fat diet-induced obesity in mice

**Fig. 2:**
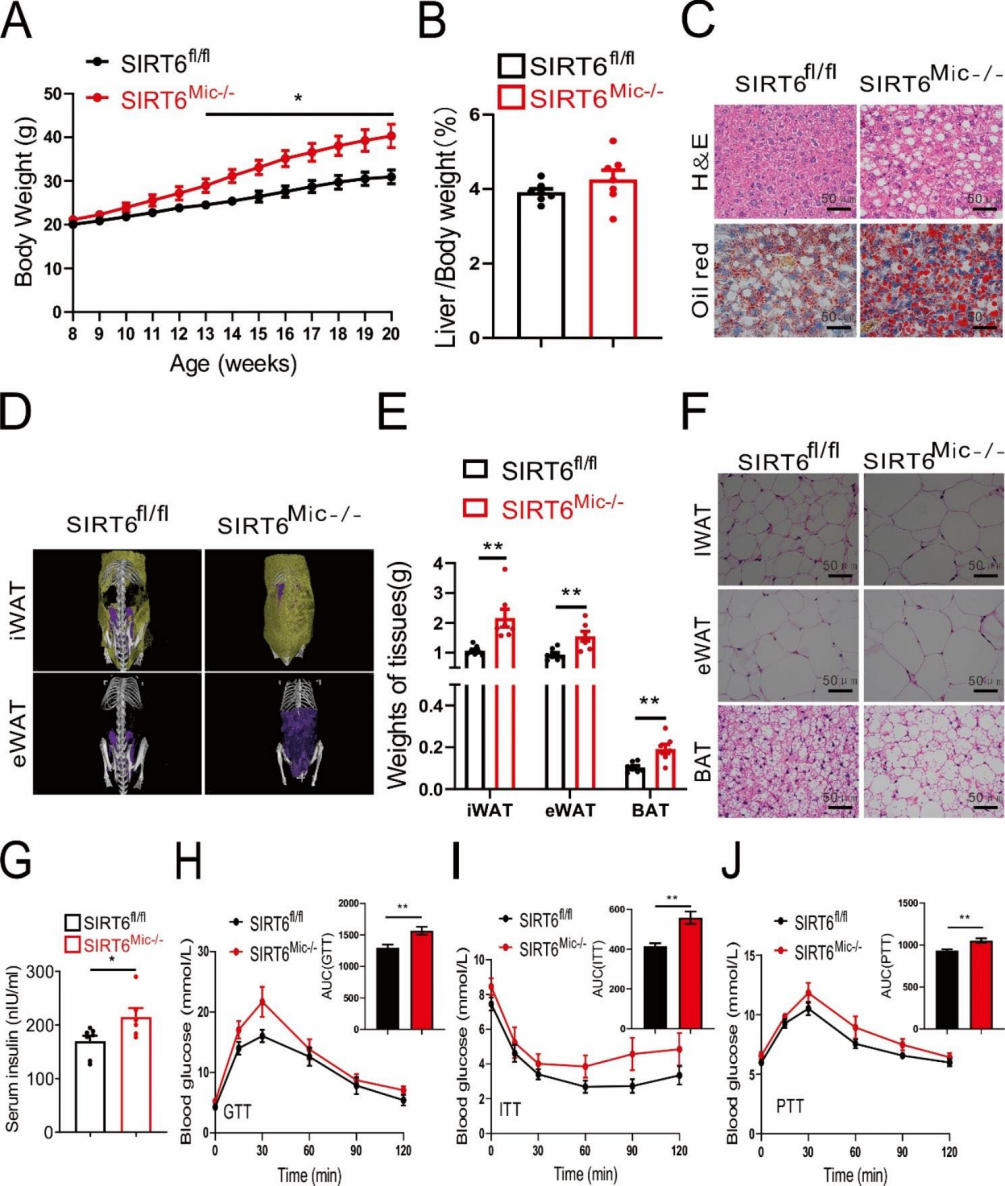
Microglia Sirt6 knockout mice are more prone to obesity after a 12-week HFD. **A** The growth curve of male mice on a HFD for 12 weeks. *n* = 7/group. **B** Liver-to-bodyweight ratio of male mice after a 12-week HFD. *n* = 7/group. **C** HE and oil red stain of liver cells of male mice after a 12-week HFD. Scale bars: 50 μm. **D** MicroCT image of iWAT and eWAT of male mice after a 12-week HFD. **E** Tissue weight of iWAT, eWAT, and BAT of male mice after a 12-week HFD. *n* = 7/group. **F** HE staining of iWAT, eWAT, and BAT of male mice after a 12-week HFD. Scale bars: 50 μm. **G** Serum insulin level in overnight fasted male mice after a 12-week HFD. *n* = 6–7/group. **H** Glucose tolerance test result of male mice after a 12-week HFD. *n* = 7/group. **I** Insulin tolerance test result of male mice after a 12-week HFD. *n* = 7/group. **J** Pyruvate tolerance test result of male mice after a 12-week HFD. *n* = 7/group. Data are presented as mean ± SEM, * *p* < 0.05, ** *p* < 0.01

To explore the effects of microglia Sirt6 on body weight and glucose homeostasis of mice fed with HFD, we measured indicators of body fat and glucose metabolism. First, SIRT6^Mic−/−^ mice were produced by mating SIRT6 ^fl/fl^ mice with Cx3cr1-Cre mice. The result of the knockout was confirmed by gene identification (Additional file 1: S1A) and fluorescence microscopy (Additional file 1: S1B). Knockout of microglia Sirt6 resulted in a significant increase in the body weight of mice after a 12-week HFD (Fig. 2A). Similarly, the liver-to-bodyweight ratio of microglia Sirt6 knockout mice slightly increased after a 12-week HFD, but the difference was negligible (Fig. 2B). As predicted, SIRT6^Mic−/−^ mice after a 12-week HFD exhibited significantly greater hepatic lipid accumulation and worse hepatic steatosis (Fig. 2C). Additionally, the adipose tissue of mice after a 12-week HFD also increased substantially, which was confirmed through the MicroCT images of inguinal white adipose tissue and epididymal white adipose tissue (iWAT and eWAT; Fig. 2D), different adipose tissue weights (Fig. 2E), and HE staining of the tissue cells (Fig. 2F). In the overnight fasted SIRT6^Mic−/−^ group, insulin levels significantly increased (Fig. 2G). Moreover, GTT, ITT, and PTT results of mice after a 12-week HFD indicated abnormal glucose homeostasis (Fig. 2H-J). In comparison, in the standard chow diet, the body weight of microglia Sirt6 knockout mice slightly increased, but the difference was negligible (Additional file 2: S2A). Similarly, weights of different adipose tissue in mice mildly increased, but the differences were negligible (Additional file 2: S2B). However, HE staining of the histiocytes exhibited Sirt6 knockdown of microglia resulted in hypertrophy of iWAT (Additional file 2: S2C). Moreover, microglia Sirt6 knockout also resulted in abnormal ITT and PTT (Additional file 2: S2D-F). Thus, the data suggested that knockout of Sirt6 in microglia exacerbates high-fat diet-induced obesity in mice.

### Sirt6 knockout in microglia leads to leptin resistance in mice

**Fig. 3:**
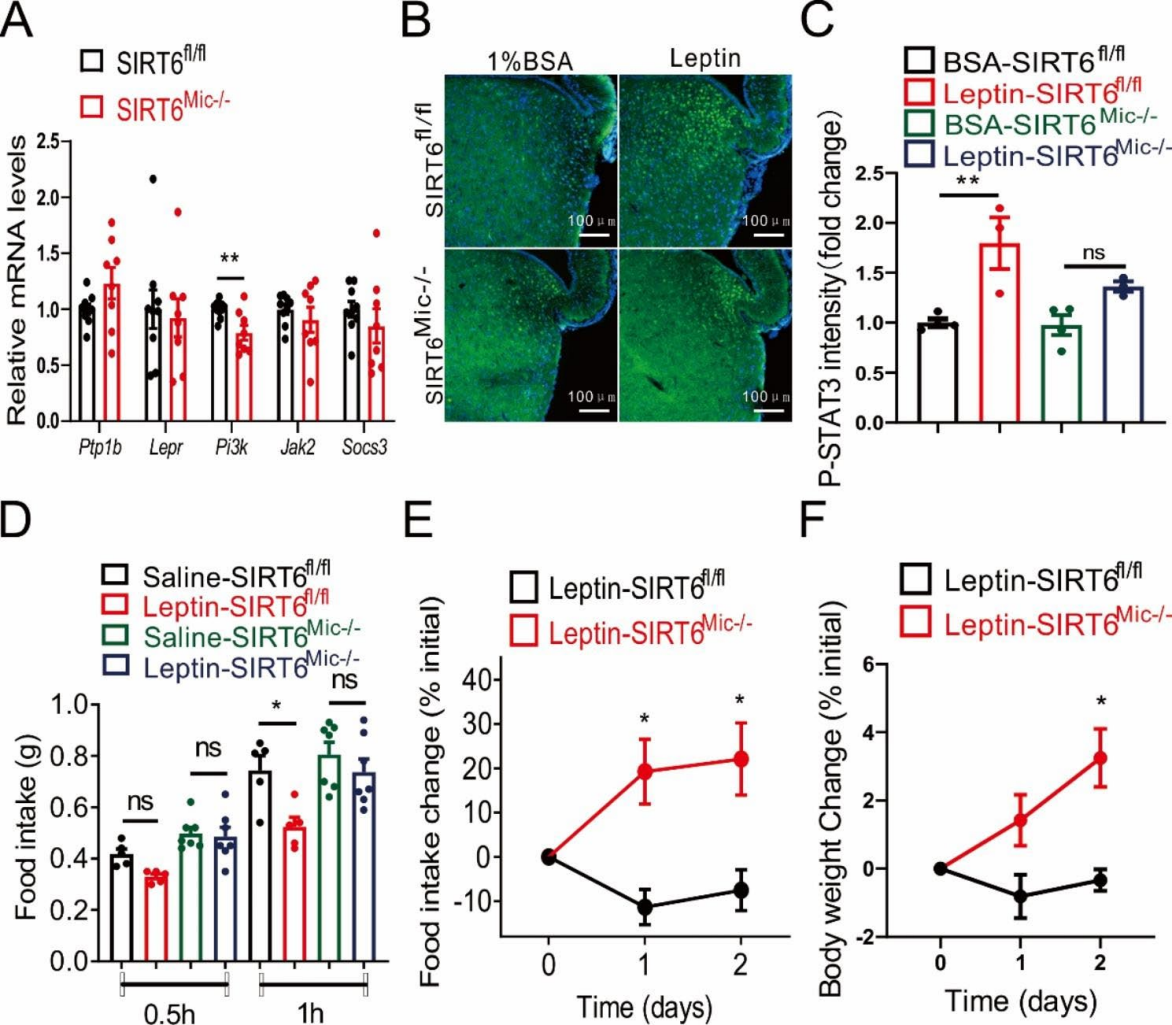
Regulation of food intake by microglia Sirt6 correlates with leptin function. **A** mRNA expression of leptin signalling in the hypothalamus of male mice under a HFD for 8 weeks. *n* = 8–9/group. **B** Fluorescence images of P-STAT3 (Ser727) in the hypothalamus of mice after injection of 1% BSA or leptin (1 µg/g) for 45 min. Scale bars: 100 μm. **C** Quantitative analysis of immunofluorescent intensity of P-STAT3 (Ser727) in the hypothalamus of mice after injection of 1% BSA or leptin (1 µg/g) for 45 min. *n* = 3–4/group. **D** Food intake change 1 h after leptin injection. *n* = 5–7/group. **E** Food intake change 2 days after leptin injection. *n* = 5/group. **F** Changes in body weight after leptin injection. *n* = 5/group. **C, D** Two-way ANOVA analysis followed by the Tukey post hoc test were performed. Data are presented as mean ± SEM, * *p* < 0.05, ** *p* < 0.01

To determine the effect of Sirt6 knockout on leptin function, *Ptp1b*, *Lepr*, *Pi3k*, *Jhak2*, and *Socs3* were selected as biomarkers (Zabolotny et al. 2002). The results showed that the expression of the *Ptp1b* gene rose, while the expression of the *Pi3k* gene fell substantially, which implied that leptin-mediated appetite-suppressing function was altered (Fig. 3A). Subsequently, we injected 1% BSA or leptin into mice and observed the fluorescence images of P-STAT3 (Ser727) in the hypothalamus. We observed inhibited activation of P-STAT3 (Ser727) in the SIRT6^Mic−/−^ mice hypothalamus, which suggested that knockout of Sirt6 inhibited the function of leptin (Fig. 3B). This finding was also confirmed by the quantitative analysis of immunofluorescent intensity of P-STAT3 (Ser727) (Fig. 3C). Next, we determined the effect of Sirt6 knockout on food intake and body weight. Sirt6 knockout eliminated leptin-mediated food intake resistance (Fig. 3D), while food intake and body weight were noticeably higher than in the control groups (Fig. 3E-F). Collectively, the results indicated that knockout of microglia Sirt6 led to leptin resistance.

### Microglia Sirt6 deficiency leads to reduced energy expenditure in high-fat diet-fed mice

**Fig. 4:**
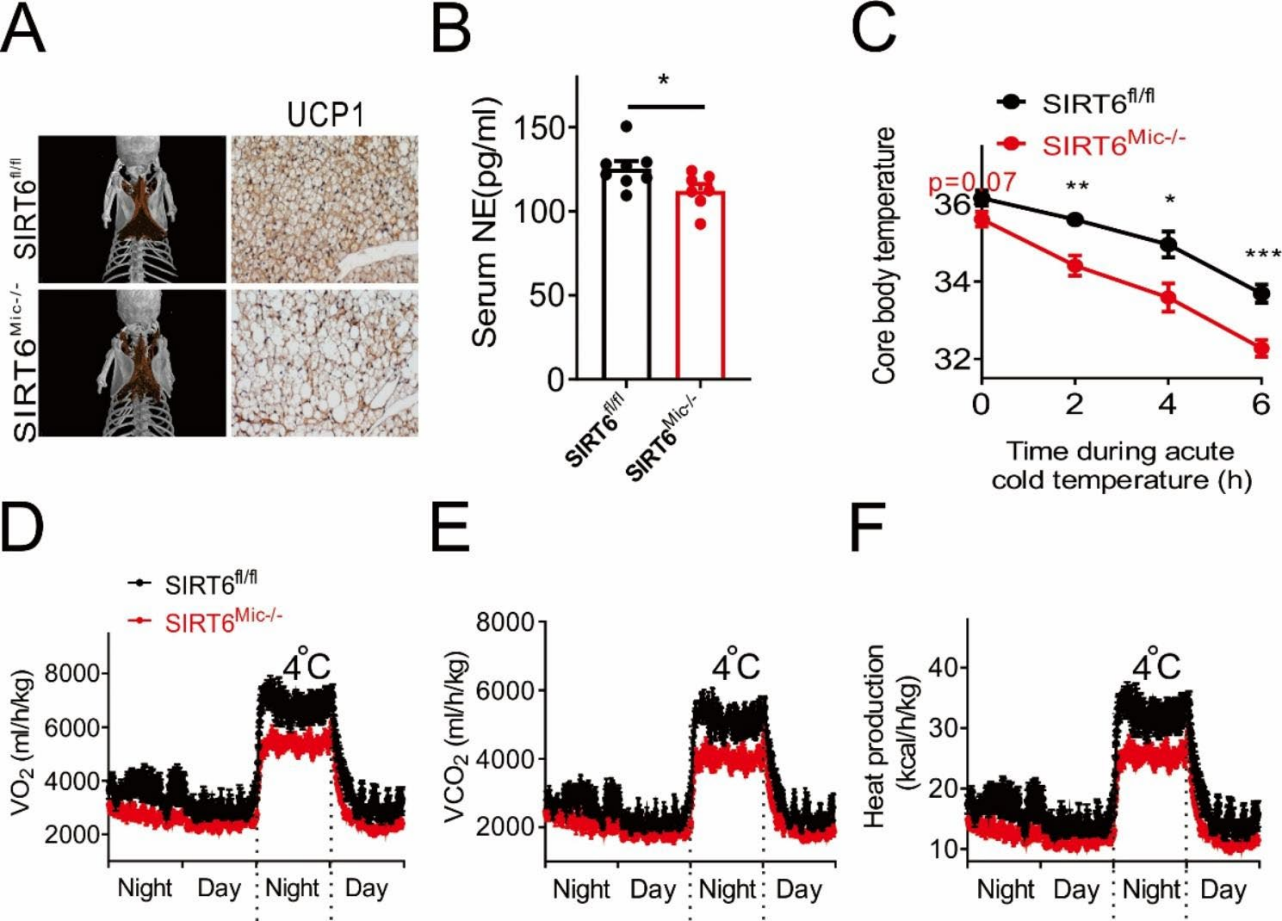
Energy expenditure in high-fat diet-fed mice with Sirt6 deficiency in microglia. **A** Images of brown fat and UCP1 immunohistochemical staining. **B** Serum NE content. *n* = 7–8/group. **C** Body core temperature of mice during acute cold exposure. *n* = 7 /group. **D** Oxygen consumption of mice exposed to acute cold temperature. *n* = 5–6/group. **E** CO_2_ production of mice exposed to acute cold temperature. *n* = 5–6/group. **F** Heat production of mice exposed to acute cold temperature. *n* = 5–6/group. Data are presented as mean ± SEM, * *p* < 0.05, ** *p* < 0.01, *** *p* < 0.001

**Fig. 5:**
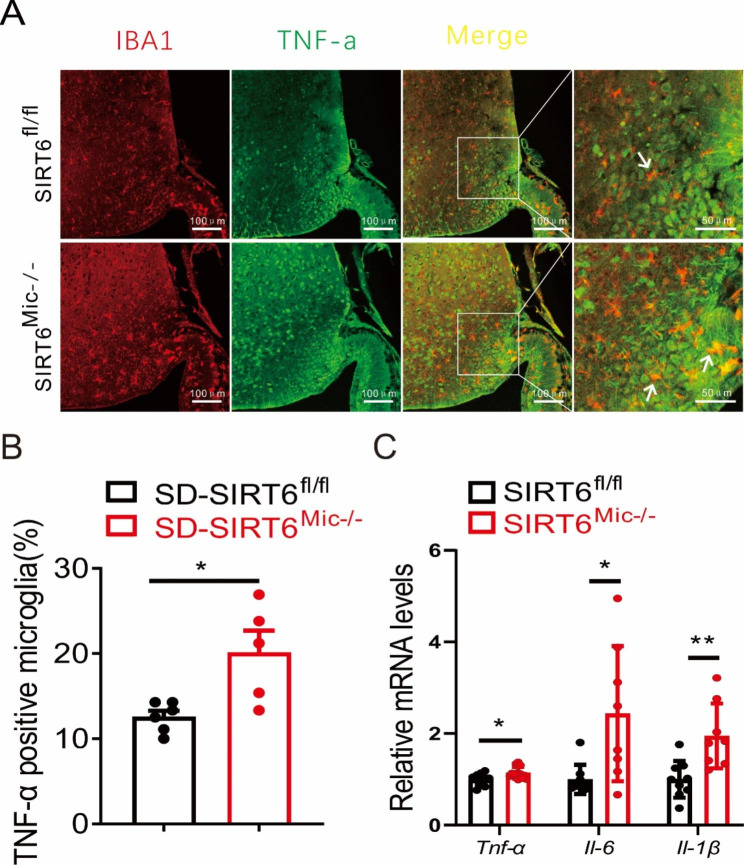
Sirt6 deficiency in microglia exacerbates high-fat diet-induced hypothalamic inflammation. **A** Immunofluorescent localization of Iba1 (red) and TNF-α (green) in hypothalamus. Arrowheads indicate representative cells showing co-localization. Scale bars: 100 μm. **B** TNF-α positive microglia counting from mice on a HFD for 12 weeks. *n* = 5–6/group. **C** mRNA expression of inflammatory factors in the hypothalamus of male mice on a HFD for 8 weeks. *n* = 8–12/group. Data are presented as mean ± SEM, * *p* < 0.05, ** *p* < 0.01

We further explored the effect of Sirt6 knockout on thermogenesis and body temperature in mice with a HFD. Sirt6 knockdown of microglia resulted in an increased volume but ‘albino’ morphology of brown adipose tissue in mice. Besides, the expression of UCP1-positive brown adipose cells decreased (Fig. 4A) and the norepinephrine content in serum fell significantly (Fig. 4B). However, in the standard chow diet, norepinephrine content remained constant (Additional file 3: S3A). As anticipated, the core body temperature of the Sirt6 knockout group was lower than the control group at the beginning and it decreased further when the mice were exposed to acute cold temperature (Fig. 5C). During this exposure, oxygen consumption, CO_2_ production, and heat production of the mice also dropped significantly (Fig. 5D-F). However, there was no significant difference in the respiratory exchange ratio (RER) of the Sirt6 knockout and control groups, indicating that the decrease was not induced by locomotor activity (Additional file 3: S3B).

### Sirt6 deficiency in microglia exacerbates high-fat diet-induced hypothalamic inflammation in mice

A HFD triggers hypothalamic inflammatory response before the body weight changes, so we further explored the effect of Sirt6 knockout on high-fat diet-induced hypothalamic inflammatory response. Immunofluorescent localization results indicated that TNF-α was highly expressed in microglial cells (Fig. 5A). This finding was also confirmed by the statistical counting of TNF-α positive microglia (Fig. 5B). Moreover, Sirt6 knockout led to increased mRNA expression of *Tnf-α, Il-6 and Il-1β* in hypothalamus of male mice after a 8-week HFD (Fig. 5C). In comparison, for the standard diet, no significant differences in hypothalamic microglial number and size were observed between SIRT6^fl/fl^ and SIRT6^Mic−/−^ mice (Additional file 4: S4A). Moreover, no significant differences in mRNA expression of inflammatory factors were observed between SIRT6^fl/fl^ and SIRT6^Mic−/−^ mice (Additional file 4: S4B). Thus, the data suggested that Sirt6 deficiency in microglia exacerbated high-fat diet-induced hypothalamic inflammation.

### Sirt6 overexpression ameliorates inflammation in BV2 cells

**Fig. 6:**
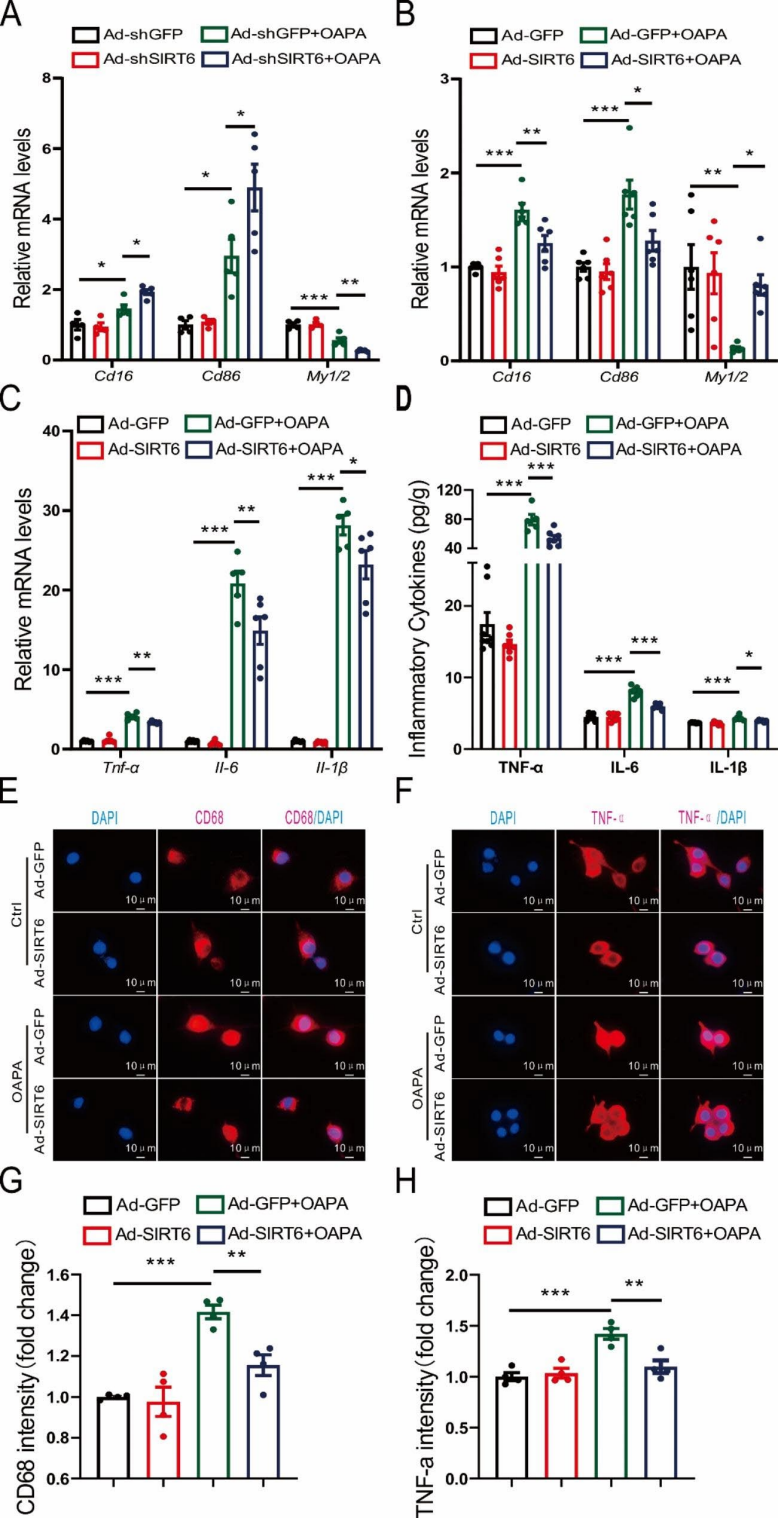
Effect of Sirt6 on the inflammation of BV2 cells treated with OA&PA for 24 h. **A** Expression of M1-type microglia surface markers (*Cd16, Cd86*) and M2-type microglia surface markers (*Ym1/2*) in BV2 cells while Sirt6 was knocked down. **B** Expression of M1-type microglia surface markers (*Cd16, Cd86*) and M2-type microglia surface markers (*Ym1/2*) in BV2 cells while Sirt6 was over-expressed. **C** mRNA levels of *Tnf-α*, *Il-6*, and *Il-1β* in BV2 cells while Sirt6 was over-expressed. **D** Inflammatory cytokine (TNF-α, IL-6, and IL-1β) content in culture medium while Sirt6 was over-expressed. **E, F** Immunofluorescence images of CD68 and TNF-α while Sirt6 was over-expressed. Scale bars: 10 μm. **G, H** Quantitative analysis of immunofluorescent intensity of CD68 and TNF-α while Sirt6 was over-expressed. **A-D, G-H** Two-way ANOVA analysis was performed, followed by the Tukey post hoc test. *n* = 4–8/group. Data are presented as mean ± SEM, * *p* < 0.05, ** *p* < 0.01, *** *p* < 0.001

To investigate the regulation of Sirt6 on microglia polarity and its effect on inflammation, we measured the changes in microglial M1/M2 polarity surface markers and inflammation cytokines in BV2 cells. CD16, CD86, and CD68 were selected as biomarkers of M1 polarization, while YM1/2 were chosen as the biomarkers of M2 polarization. Besides, TNF-α, IL-6, and IL-1β were chosen as the inflammatory cytokines (Balkwill et al. 2009; Tanaka et al. 2014; Tang et al. 2018; Zheng et al. 2020; Lopez-Castejon and Brough 2011). First, the results of sirt6 overexpression and sirt6 knockdown in BV2 cells were confirmed by Quantitative Real-Time PCR (Additional file 5: S5A-B). The results showed that the downregulation of Sirt6 led to the M1 polarization state of BV2 cells (Fig. 6A). Conversely, overexpression of Sirt6 resulted in the M2 polarization state of BV2 cells (Fig. 6B). In BV2 cells, OA&PA treatment dramatically increased the mRNA expression levels of inflammatory factors, while overexpression of Sirt6 significantly reduced their expression (Fig. 6C). These results were also confirmed by the content of inflammatory cytokines in the culture medium (Fig. 6D) and immunofluorescence images (Fig. 6E-H).

### Sirt6 overexpression significantly increases NRF2 expression in BV2 cells

**Fig. 7:**
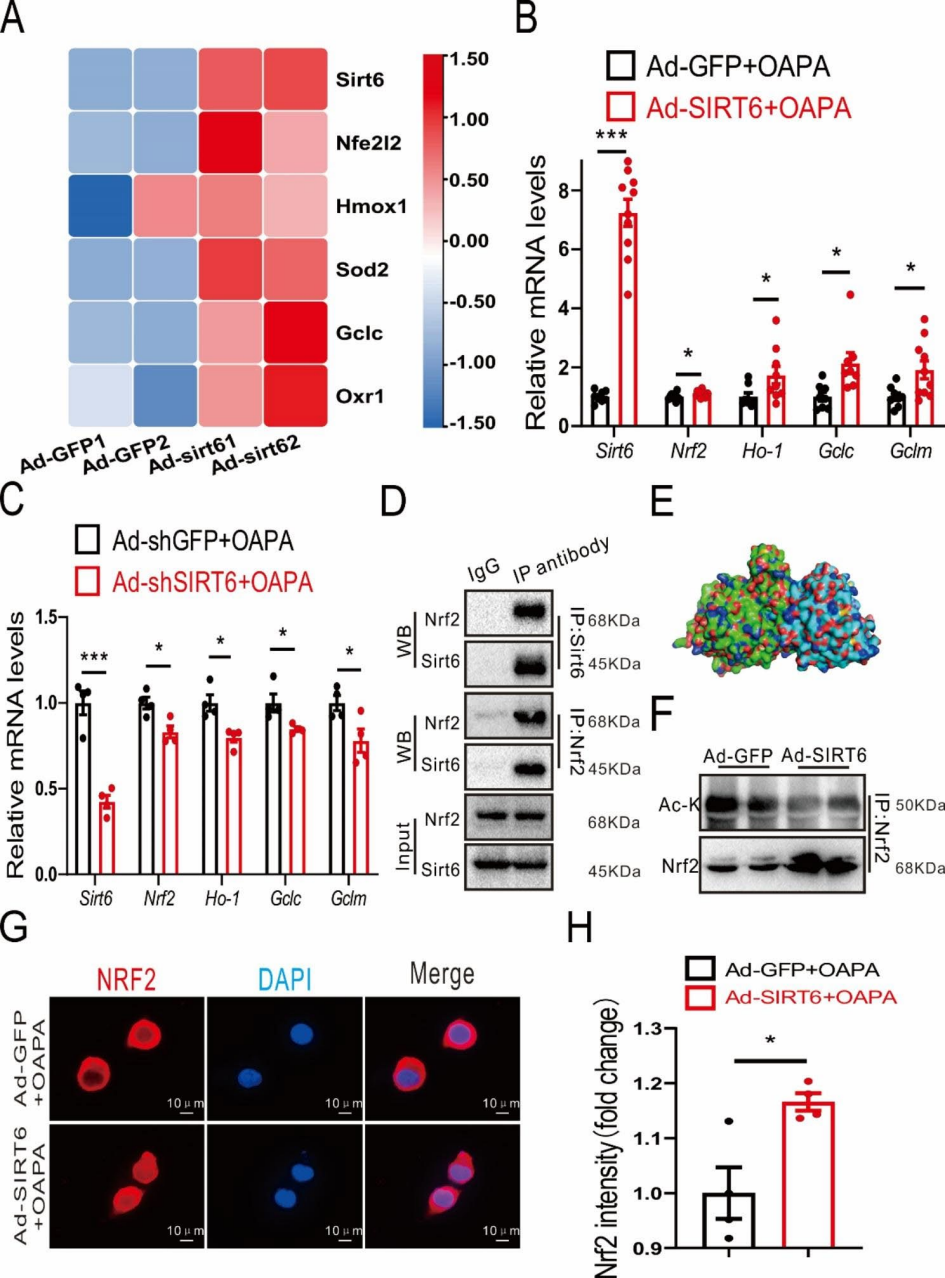
Interaction of Sirt6 with NRF2. **A** Transcriptome screening for antioxidation. *n* = 2/group. **B** Overexpression of Sirt6 increases mRNA expression of antioxidant genes *Nrf2*, *Ho-1*, *Gclc*, and *Gclm* in BV2 Cells. *n* = 8–12/group. **C** Knockdown of Sirt6 decreases mRNA expression of antioxidant genes *Nrf2*, *Ho-1*, *Gclc*, and *Gclm* in BV2 Cells. *n* = 4/group. **D** Co-IP results verify that Sirt6 interacts with NRF2 in BV2 Cells. **E** Molecular docking of Sirt6 and NRF2 protein. **F** Effect of Sirt6 overexpression on NRF2 in BV2 Cells. **G** Immunofluorescence images of NRF2 while Sirt6 was over-expressed in BV2 Cells. Scale bars: 10 μm. **H** Quantitative analysis of immunofluorescent intensity of NRF2 while Sirt6 was over-expressed in BV2 Cells. *n* = 4/group. Data are presented as mean ± SEM, * *p* < 0.05, *** *p* < 0.001

We further studied the mechanism of the Sirt6 effect on the expression of inflammatory factors. Differential expression analysis using the RNA-seq data revealed that the downstream signalling pathway of Sirt6 was associated with antioxidants (Fig. 7A). Correspondingly, antioxidant gene expression significantly increased in BV2 cells after overexpression of Sirt6 (Fig. 7B). Conversely, antioxidant gene expression significantly dncreased in BV2 cells after Knockdown of Sirt6 (Fig. 7C). Since *Nrf2* is a crucial gene that senses oxidation and regulates anti-oxidation (Friedmann Angeli and Meierjohann 2021), we hypothesised that *Nrf2* was a key gene in the downstream pathway of Sirt6. As forecast, the Co-IP experiment demonstrated that Sirt6 interacted with NRF2 in BV2 cells (Fig. 7D). Meanwhile, molecular docking showed that Sirt6 and NRF2 had binding sites (Fig. 7E), and overexpression of Sirt6 significantly increased the expression of NRF2 in BV2 cells (Fig. 7F). Moreover, immunofluorescence imaging also confirmed that Sirt6 overexpression led to an increase in NRF2 expression (Fig. 7G-H). Sirt6 is a member of the SIRT family (class III HDAC), which are highly conserved NAD+-dependent deacetylases. We constructed a Sirt6-overexpressing virus with deficient HDAC enzyme activity to verify whether the loss of HDAC enzyme activity influenced the anti-inflammatory and antioxidant effects of Sirt6. The results showed that overexpression of Sirt6 without HDAC enzyme activity eliminated the effect of Sirt6 on Nfκb-p65 and TNF-α (Additional file 6: S6A-B), as well as the antioxidant genes *Gclm* and *Gclc* (Additional file 6: S6C-D).

### NRF2 antagonists counteract Sirt6 function in BV2 cells

**Fig. 8:**
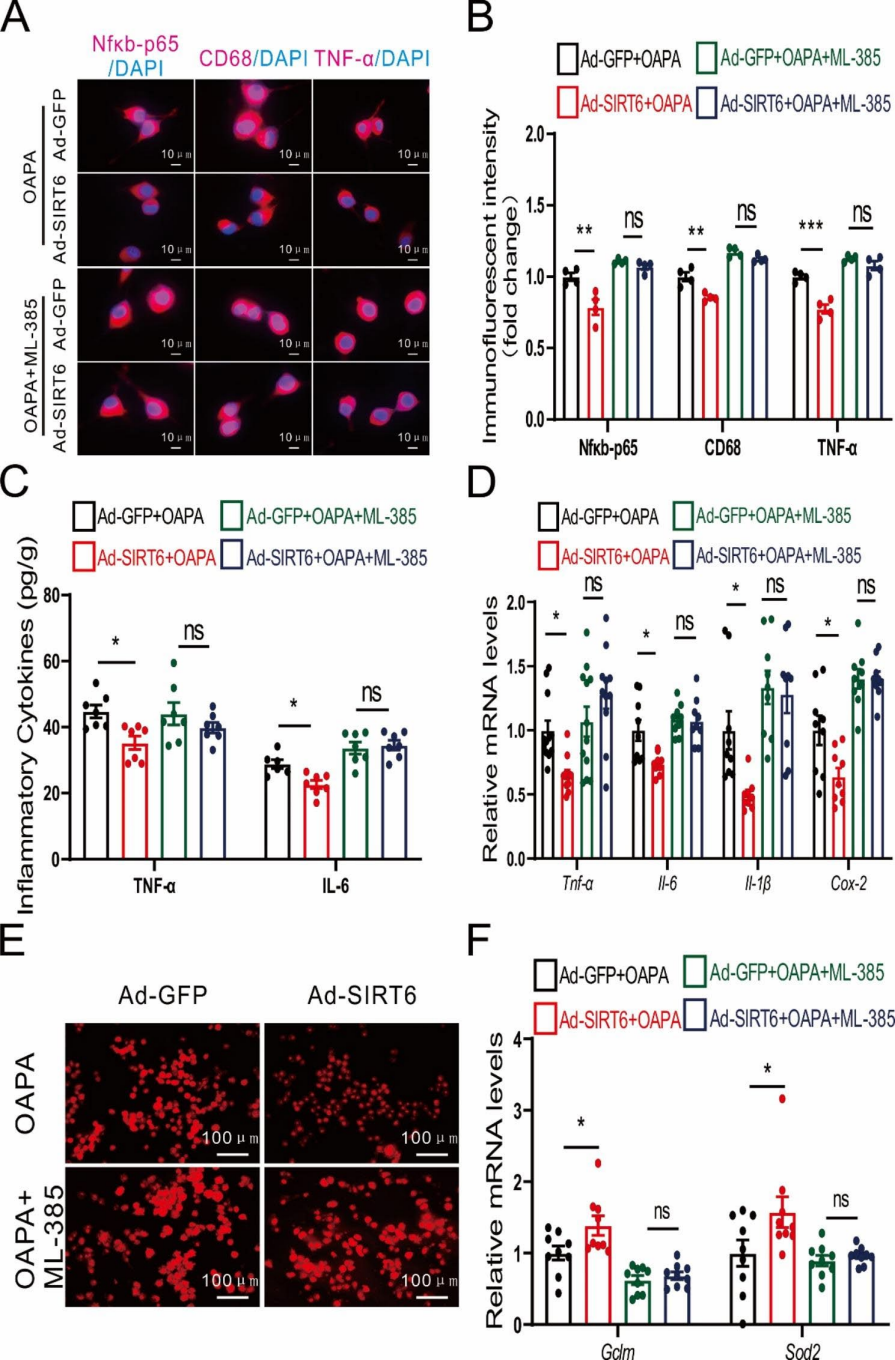
NRF2 antagonists counteract the ameliorating effects of Sirt6 on inflammation and oxidative stress. **A** Immunofluorescence images of Nfκb-p65, CD68, and TNF-α in BV2 cells with combined Sirt6 overexpression and NRF2 antagonist treatment. Scale bars: 10 μm. **B** Quantitative analysis of immunofluorescent intensity of Nfκb-p65, CD68, and TNF-α in BV2 cells with combined Sirt6 overexpression and NRF2 antagonist treatment. *n* = 4/group. **C** Expression of inflammatory cytokines TNF-α and IL-6. *n* = 6–7/group. **D** mRNA expression of *Tnf-α*, *Il-6*, *Il-1β*, and *Cox-2*. *n* = 9–12/group. **E** Immunofluorescence images of ROS. Scale bars: 100 μm. **F** mRNA expression levels of *Gclm* and *Sod2*. *n* = 9/group. **B-D, F** Two-way ANOVA analysis followed by the Tukey post hoc test were performed. ns: not significant. Data are presented as mean ± SEM, * *p* < 0.05, ** *p* < 0.01, *** *p* < 0.001

To further verify that Sirt6 reduced the expression of inflammatory factors through the NRF2 pathway, we used antagonists to inhibit NRF2 and measured the effect of Sirt6 on inflammatory gene expression. Immunofluorescence results showed that after ML-385 blocked NRF2 expression, overexpression of Sirt6 had no significant effect on the expression of Nfκb-p65, CD68, and TNF-α (Fig. 8A-B). Furthermore, inflammatory cytokine content and mRNA expression of inflammatory genes exhibited no significant difference (Fig. 8C-D). Subsequently, we further determined the ROS content. As anticipated, ROS content showed no significant difference in the Sirt6 overexpression group after NRF2 was blocked (Fig. 8E). Moreover, mRNA expression of *Gclm* and *Sod2* showed a negligible difference in the Sirt6 overexpression group after NRF2 antagonist treatment (Fig. 8F).

## Discussion

Microglia maintain synaptic remodelling, neurogenesis, and elimination of unwanted neurons and cellular debris, thereby promoting inner brain tissue homeostasis and regulating energy metabolism. This balance can be interrupted by high-fat diet-induced chronic inflammation. However, the specific mechanism of this process remains unknown (Wang et al. 2021). In this study, we found that Sirt6 was crucial in maintaining microglia function. Sirt6 ameliorated long-term high-fat diet-induced obesity, altered microglia polarity, and alleviated the inflammatory response in the hypothalamus. These processes were accomplished by the deacetylation of NRF2 by Sirt6 in microglia.

A HFD can cause a state of inflammation occurs in the hypothalamus (Alexaki 2021). As the resident immune cells of the brain, microglia transform into pro-inflammatory (M1) phenotype in the context of HFD. Inhibiting microglia expansion hinders diet-induced body weight gain while preventing hypothalamic and peripheral inflammation due to caloric overload (André et al. 2017). Several studies have demonstrated that the transition of microglia from M1 to M2 can be mediated by multiple mechanisms. For instance, M2 microglia polarization is promoted by SIRT1, which reduces ROS-mediated NLRP3 inflammasome signalling (Xia et al. 2021). Existing research has proved that Sirt6 eliminated inflammatory response in the brian, as mentioned above (He et al. 2021). In this study, we first explored the effect of Sirt6 in protecting microglia. Our data showed that long-term HFD reduced Sirt6 expression of microglia in the hypothalamic region. Knockout of microglia Sirt6 exacerbated high-fat diet-induced hypothalamic microglial activation and inflammation. Conversely, further experiments in vitro proved that Sirt6 overexpression shifted microglia polarity and moderated inflammation. Collectively, Our data suggested that Sirt6 may have a key role in protecting microglia.

Furthermore, leptin is an adipocyte-derived hormone that contributes to the homeostatic regulation of energy balance and metabolism through humoral and neural pathways (Liu et al. 2018). Previous studies have shown that the basic mechanisms related to leptin resistance include: blood-brain barrier transporter disorder, competitive inhibition of leptin, mutations of LepR, and impairment of leptin cellular signalling (Liu et al. 2018). Here, we demonstrated that knockout of Sirt6 in microglia resulted in ineffective leptin-mediated appetite suppression and weight gain in mice. Similarly, Tang et al. found that Sirt6 in pro-opiomelanocortin (POMC) neurons controlled energy metabolism by modulating leptin signalling (Tang et al. 2020). HFD has a significant effect on the cytoarchitecture of the arcuate nucleus, which may be irreversible due to reactive gliosis (Horvath et al. 2010). Our results revealed that knockout of Sirt6 in microglia led to leptin resistance, which further confirmed Sirt6 as a protector of microglia.

In addition to suppressing appetite, Sirt6 also plays a key role in regulating fat thermogenesis (Kuang et al. 2018). In adipose tissue, Sirt6 deletion moderates the binding of phosphorylated ATF2 to the PGC-1α promoter, which subsequently reduces the thermogenic programme in brown fat and eventually leads to obesity (Yao et al. 2017). Sirt6 is an important component of the CNS, and we discovered that its loss in hypothalamic microglia led to reduced energy expenditure, which was manifested by impaired brown cell function, lower body temperature in cold environments, and reduced energy expenditure. This process in turn led to weight gain and increased the liver-to-weight ratio and hypertrophy of white adipose tissue, ultimately resulting in obesity.

Sirt6 is also involved in the regulation of oxidative stress-related diseases (Tasselli et al. 2017; Chang et al. 2020). In tissue such as liver, human mesenchymal stem cells, and human lens epithelial cells, Sirt6 cooperates with NRF2 to achieve antioxidant and anti-inflammatory effects (Zhou et al. 2021; Pan et al. 2016; Sun et al. 2019). For instance, Sirt6 cooperates with NRF2 to attenuates APAP-induced hepatotoxicity by inhibiting oxidative stress (Zhou et al. 2021). SIRT6 also protects human mesenchymal stem cells from oxidative stress via NRF2 (Pan et al. 2016). Moreover, activation of SIRT6-Nrf2 signaling protects human lens epithelial cells from ultra-violet-induced oxidative damage (Sun et al. 2019). In this study, we revealed that in microglia, Sirt6 overexpression improved inflammatory response and oxidative stress, and its mechanism was the deacetylation of NRF2. As mentioned above, NRF2 and its endogenous inhibitor, Keap1, function as a ubiquitous, evolutionarily conserved intracellular defence mechanism to counteract oxidative stress (Silva-Islas and Maldonado 2018; Bellezza et al. 2018). We found that Sirt6 deacetylated and stabilised NRF2 to increase the expression of anti-oxidative genes and defend against ROS overload. Moreover, pharmacological inhibition of NRF2 eliminated the beneficial modulating effects of Sirt6 on microglial activity. Our findings further elucidated the mechanism of the microglia Sirt6 for oxidative stress and inflammatory responses, thereby providing novel insights into how the CNS defend against obesity induced by a HFD. Collectively, this study of Sirt6 in the energy regulation of microglia complements current knowledge on the mechanism by which Sirt6 controls whole-body energy metabolism in the CNS.

## Conclusion

Our results reveal that microglia Sirt6 in the hypothalamus is essential in the regulation of metabolism and further prove that Sirt6 plays an important role in high-fat diet-induced obesity via the manipulation of hypothalamic inflammatory response and energy expenditure. Therefore, microglial Sirt6 may be an important therapeutic target for obesity.

### Electronic supplementary material

Below is the link to the electronic supplementary material.

**Additional file 1: S1** Confirmation of microglia Sirt6 knockout mice. **A** Gene identification of microglia Sirt6 knockout of mice. **B** Validation of Sirt6 knockout mice by immunofluorescence. Scale bars: 100 μm

**Additional file 2: S2** Effects of microglia Sirt6 knockout on body weight and glucose homeostasis of mice under a standard diet. **A** Body weight of Sirt6 knockout mice on a standard diet. *n* = 6/group. **B** Tissue weight of iWAT, eWAT, and BAT. *n* = 6/group. **C** HE staining of iWAT, eWAT, and BAT. Scale bars: 50 μm. **D-F** Results of GTT, ITT, and PTT tests in mice under the standard chow diet. *n* = 8/group. Data are presented as mean ± SEM, ** *p* < 0.01, ***p < 0.001

**Additional file 3: S3** Serum NE content and RER of microglia Sirt6 knockout mice. **A** Serum NE content under a standard diet. *n* = 4–5/group. **B** RER result of mice fed with HFD exposed to acute cold temperature. Data are presented as mean ± SEM

**Additional file 4: S4** Expression of hypothalamic inflammation in male mice under a standard diet. **A** Fluorescence images of Ibal in hypothalamus of male mice under a standard diet. Scale bars: 100 μm. **B** mRNA expression of inflammatory factors in hypothalamus of male mice under a standard diet. *n* = 7–11/group. Data are presented as mean ± SEM. ns: not significant.

**Additional file 5: S5** Confirmation of Sirt6 expression and knockdown. **A** mRNA expression level of *Sirt6* in BV2 cells while Sirt6 was over-expressed. **B** mRNA expression level of *Sirt6* in BV2 cells while Sirt6 was knocked down. **A, B** Welch’s t-test. *n* = 6/group. Data are presented as mean ± SEM, ** *p* < 0.01, *** *p* < 0.001

**Additional file 6: S6** Effect of Sirt6 without HDAC enzyme activity on the inflammation and antioxidation of BV2 cells treated with OA&PA for 24 h. **A** Overexpression of Sirt6 without HDAC enzyme activity eliminates the effect of Sirt6 on Nfkb-p65. Scale bars: 10 μm. **B** Overexpression of Sirt6 without HDAC enzyme activity eliminates the effect of Sirt6 on TNF-α. Scale bars: 100 μm. **C** mRNA expression level of *Gclc*. **D** mRNA expression level of *Gclm*. **C, D** One-way ANOVA analysis followed by the Tukey post hoc test were performed. *n* = 7–8/group. Data are presented as mean ± SEM, * *p* < 0.05, *** *p* < 0.001

## Data Availability

Data will be made available on request.

## Abbreviations

HFD: High-fat diet
PVN: Paraventricular nucleus
PFA: Perifornical area
LHA: Lateral hypothalamic area
ARC: Arcuate nucleus
DIO: Diet-induced obesity
CNS: Central nervous system
NRF2: Nuclear Factor Erythroid 2-related Factor 2
Keap1: Kelch-like ECH-associated Protein 1
ARE: Antioxidant Response Elements
SD: Standard chow diet
H&E: Hematoxylin and eosin
GTT: Glucose tolerance test
ITT: Insulin tolerance test
PTT: Pyruvate tolerance test
CLAMS: Comprehensive laboratory animal monitoring system
OA: Oleic acid
PA: Palmitic acid
ROS: Reactive oxygen species
POMC: Pro-opiomelanocortin
