# Correction: Microglia Sirt6 modulates the transcriptional activity of NRF2 to ameliorate high-fat diet-induced obesity

**DOI:** 10.1186/s10020-023-00723-5

**Published:** 2023-09-19

**Authors:** Xiaoxia Xiao, Huiling Hu, Yadi Zhong, Yingjian Chen, Kaijia Tang, Zhisen Pan, Jiawen Huang, Xiaoying Yang, Qi Wang, Yong Gao

**Affiliations:** 1 Science and Technology Innovation Center, Guangzhou University of Chinese Medicine, Guangzhou, 510006 China; 2 Department of Clinical Laboratory, Sun Yat-Sen Memorial Hospital, Sun Yat-Sen University, Guangzhou, 510289 China; 3 Guangdong Provincial Key Laboratory of Malignant Tumor Epigenetics and Gene Regulation, Sun Yat- Sen Memorial Hospital, Sun Yat-Sen University, Guangzhou, 510289 China; 4 First Affiliated Hospital, Guangzhou University of Chinese Medicine, Guangzhou, 510006 China; 5 Jiangsu Key Laboratory of Immunity and Metabolism, Department of Pathogen Biology and Immunology, Xuzhou Medical University, Xuzhou, 221004 Jiangsu China

**Correction: Molecular Medicine (2023) 29:108**

10.1186/s10020-023-00676-9

Following publication of the original article (Xiao et al. 2023), the authors would like to add the equal contribution to their work.

This Correction article shows the equal contribution.
