# Supplementary figures and images for: Microglia Sirt6 modulates the transcriptional activity of NRF2 to ameliorate high-fat diet-induced obesity
Source: Mol Med. 2023 Aug 15;29:108. doi: 10.1186/s10020-023-00676-9 (PMC10428617; doi:10.1186/s10020-023-00676-9)

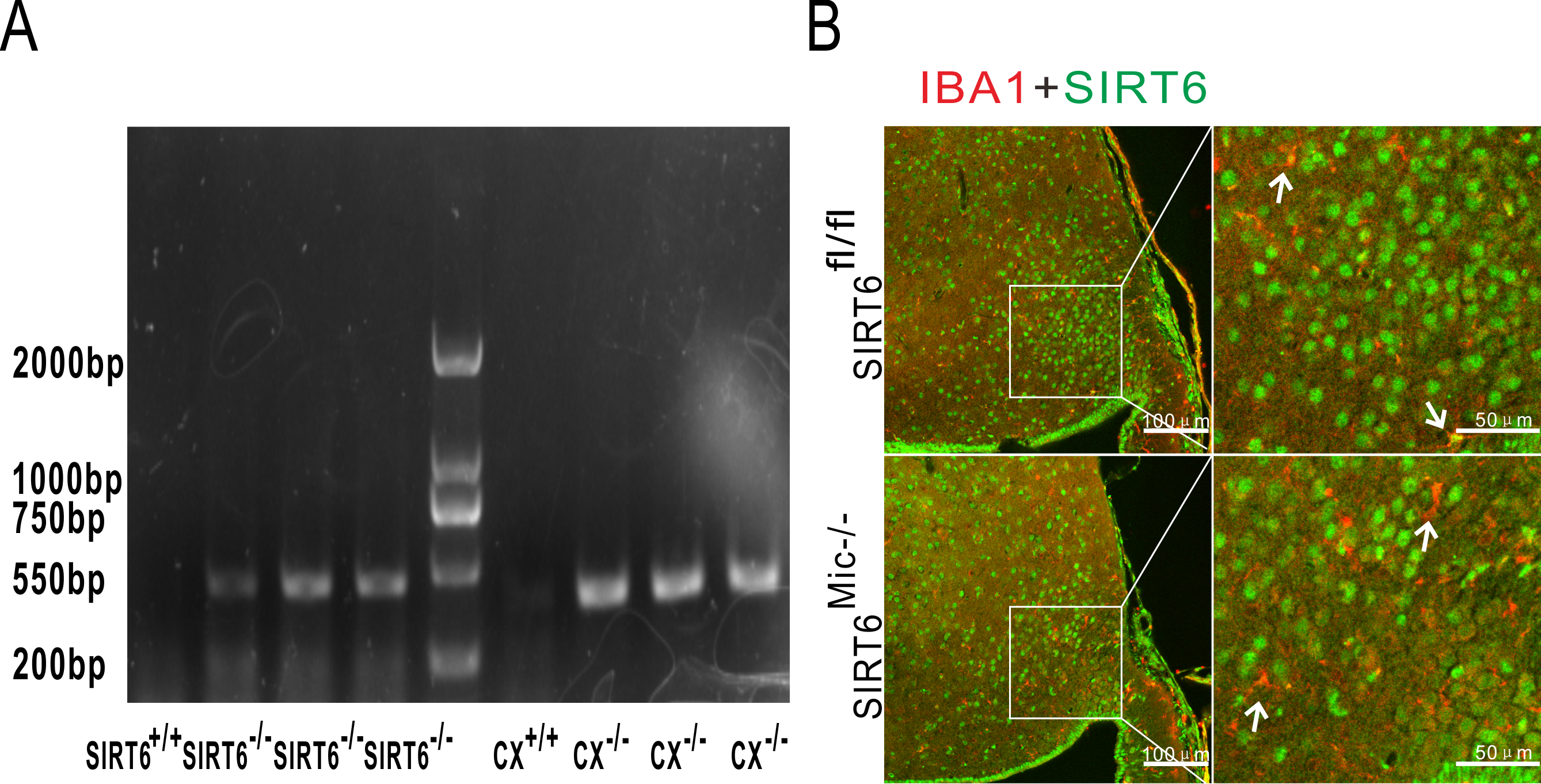

Supplement: Supplementary file 1 — Additional file 1: S1 Confirmation of microglia Sirt6 knockout mice. A Gene identification of microglia Sirt6 knockout of mice. B Validation of Sirt6 knockout mice by immunofluorescence. Scale bars: 100 μm [file 10020_2023_676_MOESM1_ESM.tif]

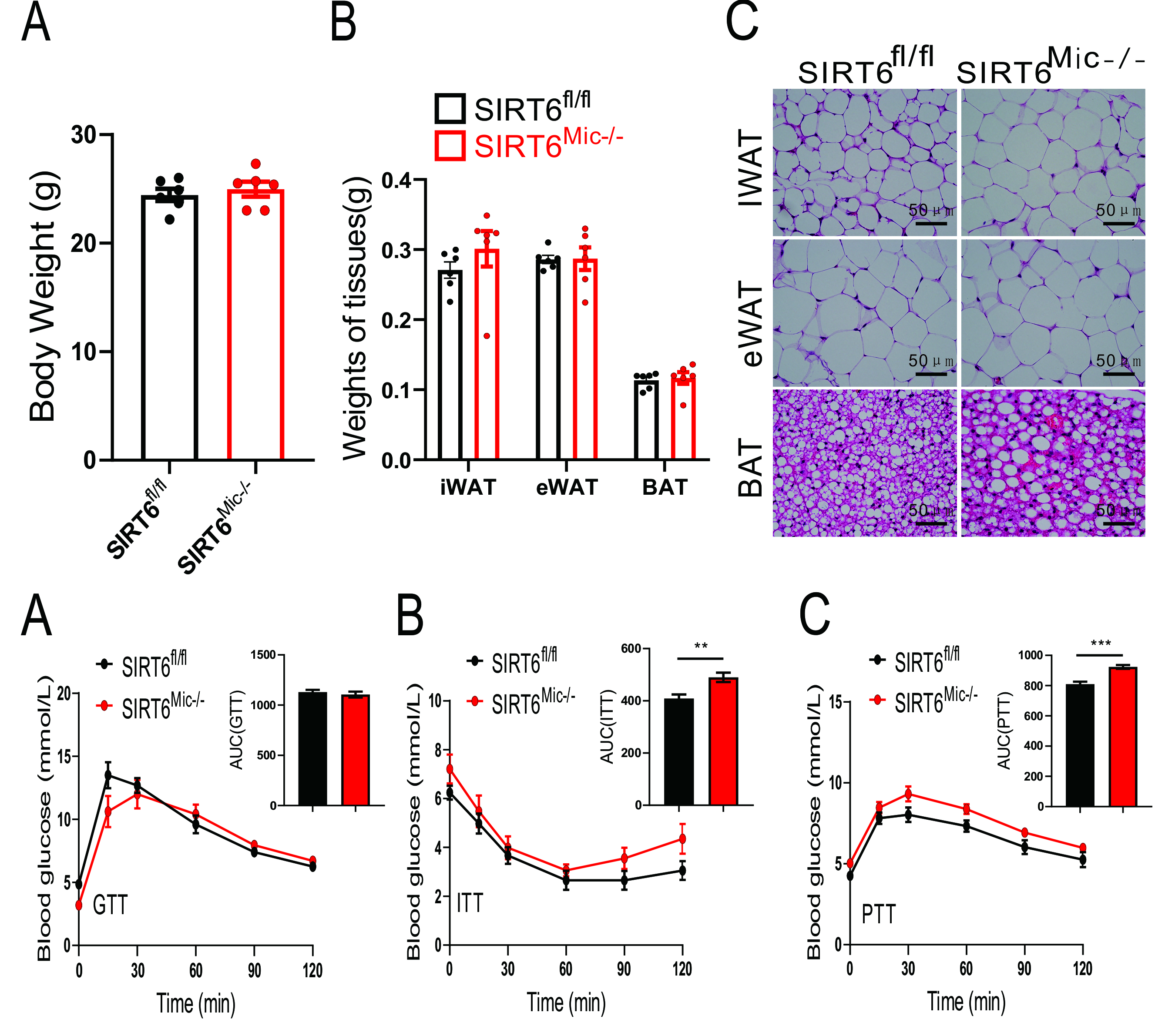

Supplement: Supplementary file 2 — Additional file 2: S2 Effects of microglia Sirt6 knockout on body weight and glucose homeostasis of mice under a standard diet. A Body weight of Sirt6 knockout mice on a standard diet. n = 6/group. B Tissue weight of iWAT, eWAT, and BAT. n = 6/group. C HE staining of iWAT, eWAT, and BAT. Scale bars: 50 μm. D-F Results of GTT, ITT, and PTT tests in mice under the standard chow diet. n = 8/group. Data are presented as mean ± SEM, ** p < 0.01, ***p < 0.001 [file 10020_2023_676_MOESM2_ESM.tif]

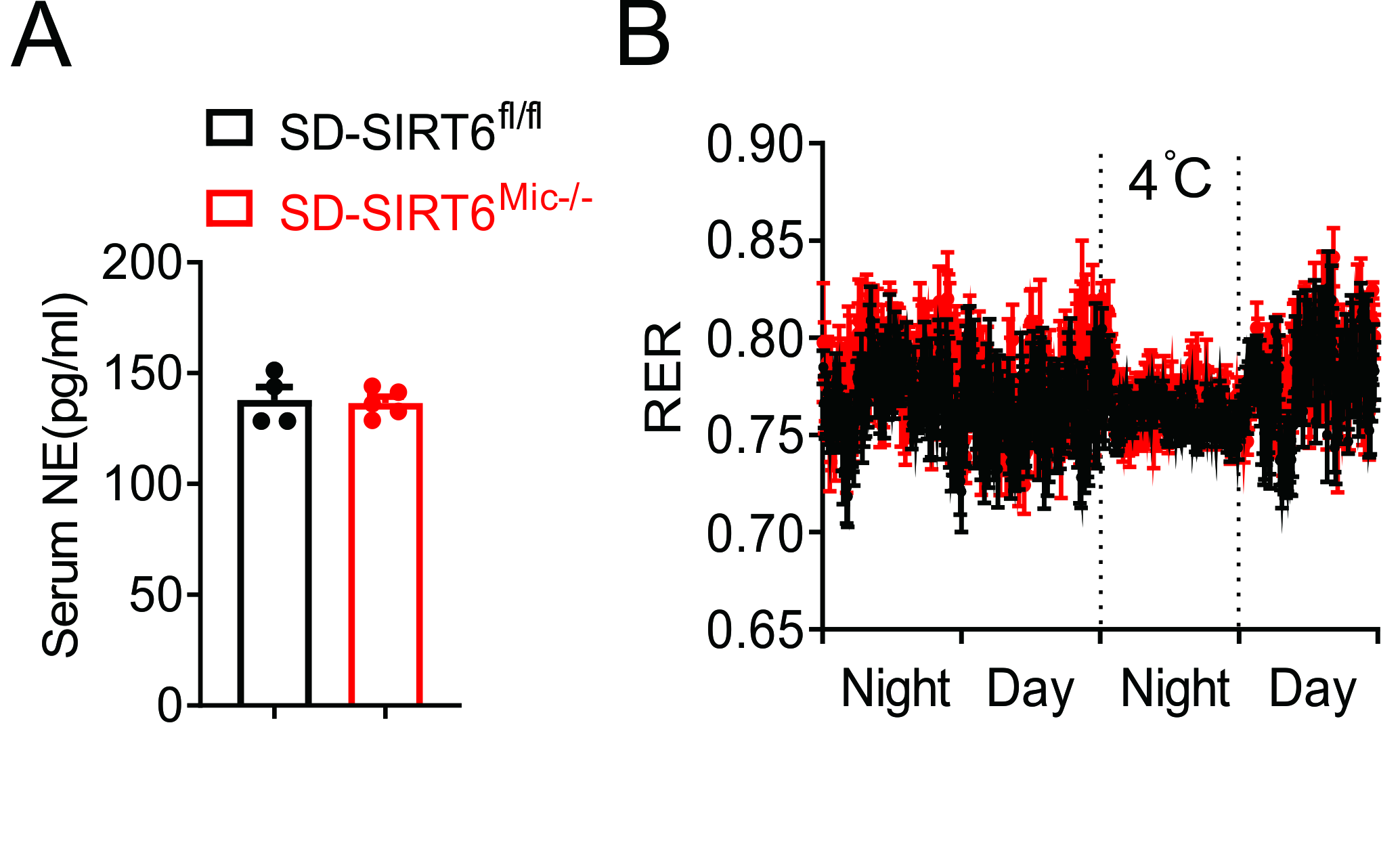

Supplement: Supplementary file 3 — Additional file 3: S3 Serum NE content and RER of microglia Sirt6 knockout mice. A Serum NE content under a standard diet. n = 4–5/group. B RER result of mice fed with HFD exposed to acute cold temperature. Data are presented as mean ± SEM [file 10020_2023_676_MOESM3_ESM.tif]

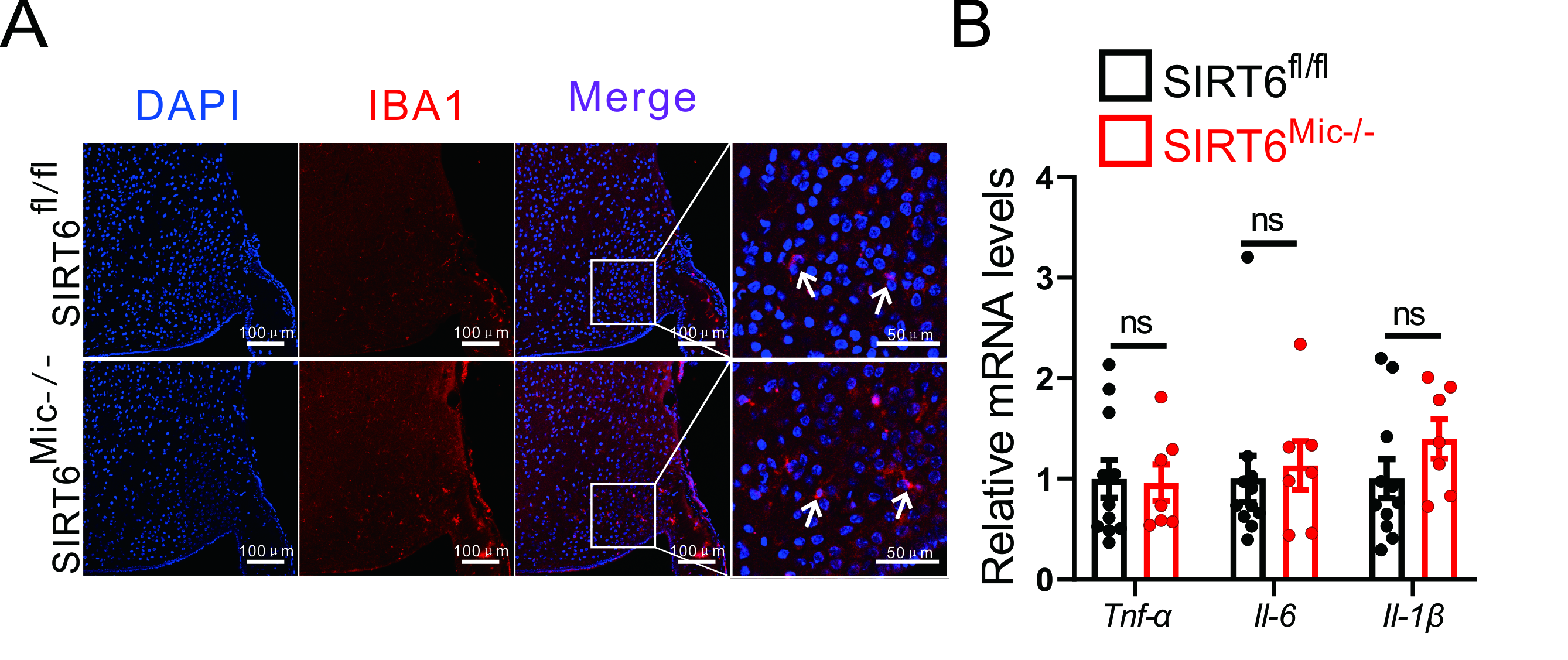

Supplement: Supplementary file 4 — Additional file 4: S4 Expression of hypothalamic inflammation in male mice under a standard diet. A Fluorescence images of Ibal in hypothalamus of male mice under a standard diet. Scale bars: 100 μm. B mRNA expression of inflammatory factors in hypothalamus of male mice under a standard diet. n = 7–11/group. Data are presented as mean ± SEM. ns: not significant. [file 10020_2023_676_MOESM4_ESM.tif]

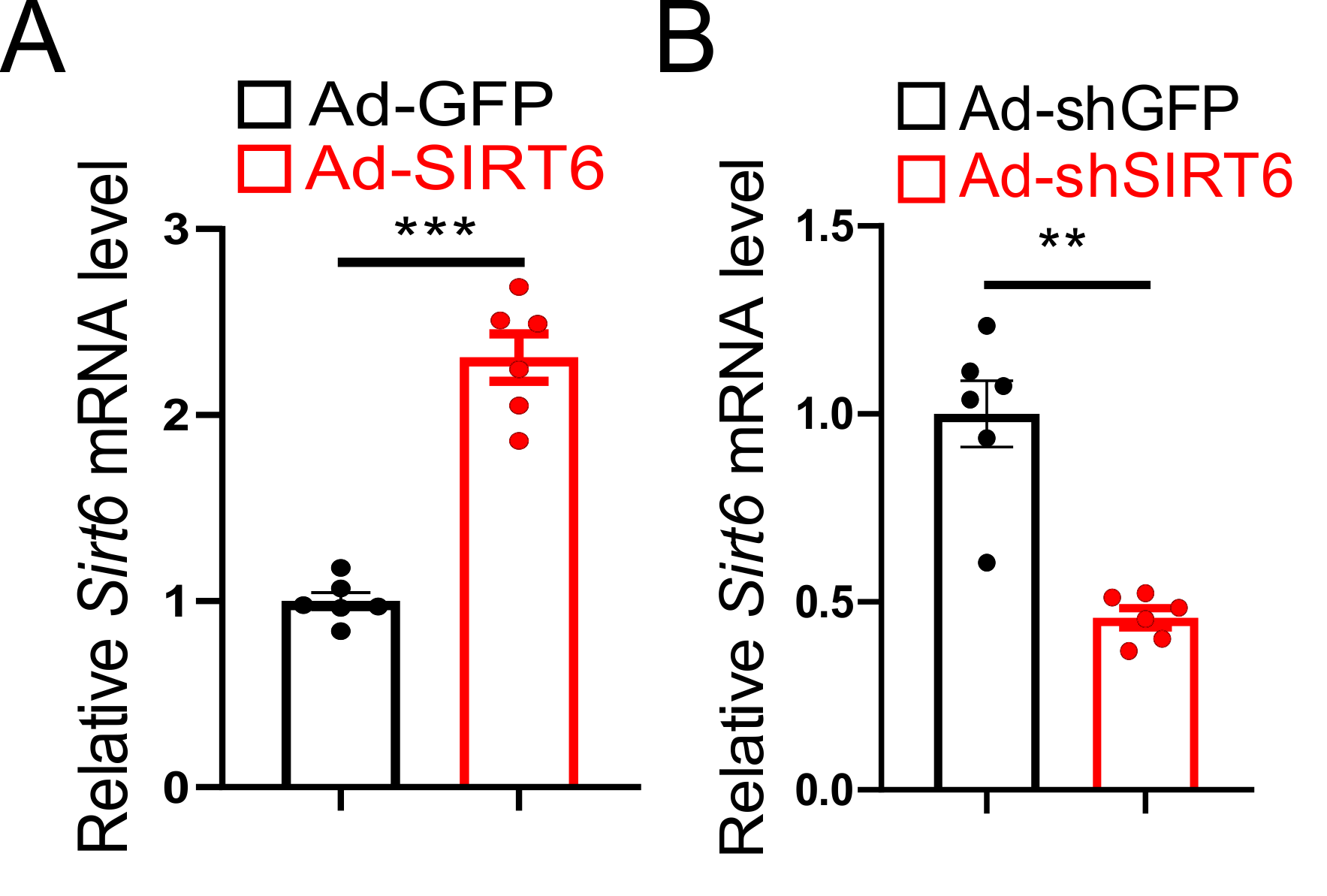

Supplement: Supplementary file 5 — Additional file 5: S5 Confirmation of Sirt6 expression and knockdown. A mRNA expression level of Sirt6 in BV2 cells while Sirt6 was over-expressed. B mRNA expression level of Sirt6 in BV2 cells while Sirt6 was knocked down. A, B Welch’s t-test. n = 6/group. Data are presented as mean ± SEM, ** p < 0.01, *** p < 0.001 [file 10020_2023_676_MOESM5_ESM.tif]

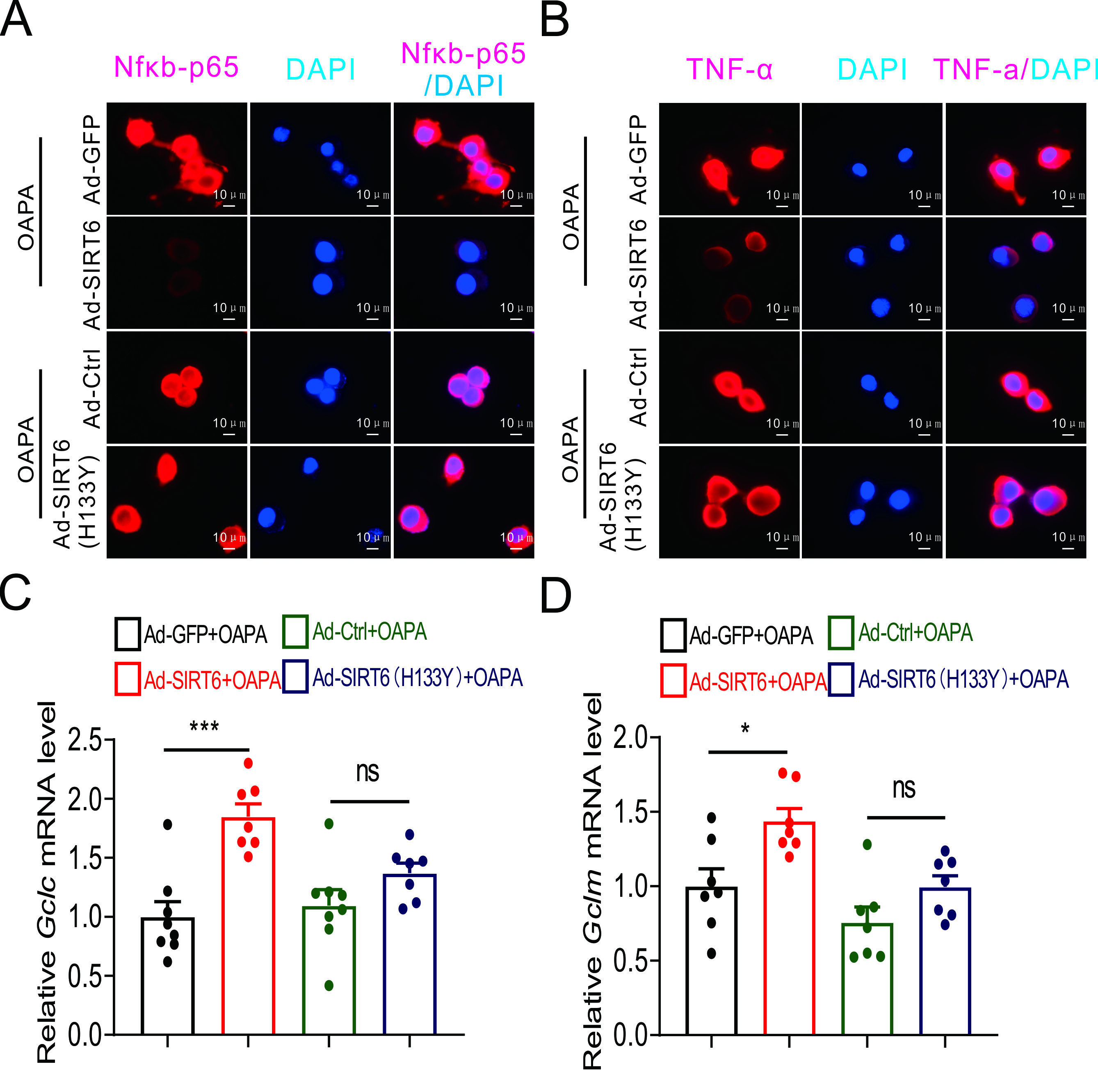

Supplement: Supplementary file 6 — Additional file 6: S6 Effect of Sirt6 without HDAC enzyme activity on the inflammation and antioxidation of BV2 cells treated with OA&PA for 24 h. A Overexpression of Sirt6 without HDAC enzyme activity eliminates the effect of Sirt6 on Nfkb-p65. Scale bars: 10 μm. B Overexpression of Sirt6 without HDAC enzyme activity eliminates the effect of Sirt6 on TNF-α. Scale bars: 100 μm. C mRNA expression level of Gclc. D mRNA expression level of Gclm. C, D One-way ANOVA analysis followed by the Tukey post hoc test were performed. n = 7–8/group. Data are presented as mean ± SEM, * p < 0.05, *** p < 0.001 [file 10020_2023_676_MOESM6_ESM.tif]
